# Tailoring Zn^2+^ Flux by an Ion Acceleration Layer Modified Separator for High‐Rate Long‐Lasting Zn Metal Anodes

**DOI:** 10.1002/advs.202407410

**Published:** 2024-10-08

**Authors:** Yicheng Tan, Duo Chen, Tengyu Yao, Yiming Zhang, Chenglin Miao, Hang Yang, Yuanhang Wang, Li Li, Volodymyr Kotsiubynskyi, Wei Han, Laifa Shen

**Affiliations:** ^1^ Jiangsu Key Laboratory of Electrochemical Energy Storage Technologies College of Materials Science and Technology Nanjing University of Aeronautics and Astronautics Nanjing 210016 China; ^2^ College of Physics State Key Laboratory of Inorganic Synthesis and Preparative Chemistry International Center of Future Science Jilin University Changchun 130012 China; ^3^ Electrochemical Innovation Lab Department of Chemical Engineering University College London Torrington Place London WC1E 7JE UK; ^4^ Material Science and Novel Technology Department Vasyl Stefanyk Precarpathian National University Ivano–Frankivsk 76018 Ukraine

**Keywords:** dendrite‐free, ion flux, separator, zinc ion battery, Zn metal anode

## Abstract

A large concentration gradient originating from sluggish ion transport on the surface of Zn metal anodes will result in uneven Zn^2+^ flux, giving rise to severe dendrite growth, especially at high current density. Herein, an ion acceleration layer is introduced by a facile separator engineering strategy to realize modulated Zn^2+^ flux and dendrite‐free deposition. Zinc hexacyanoferrate as the modifying agent featuring strong zincophilicity and rapid diffusion tunnel can enable fast trap for Zn^2+^ near the electrode surface and immediate transport onto deposition sites, respectively. The ion acceleration effect is substantiated by improved ion conductivity, decreased activated energy, and promoted Zn^2+^ transference number, which can moderate concentration gradient to guide homogenous Zn^2+^ flux distribution. As a result, the separator engineering guarantees Zn||Zn symmetrical cells with long‐term stability of 2700 h at 2 mA cm^−2^, and 1770 h at a large current density of 10 mA cm^−2^. Moreover, cycling stability and rate capability for full cells with different cathodes can be substantially promoted by the modified separator, validating its superior practical feasibility. This study supplies a new scalable approach to tailoring ion flux near the electrode surface to enable robust Zn metal anodes at a high current density.

## Graphical abstract

An ion acceleration layer is introduced by separator engineering to promote the ion transfer process, mitigate ion concentration gradient, and guide homogenous Zn^2+^ flux at high‐current density, which realizes prolonged dendrite‐free Zn metal anode. Zinc hexacyanoferrate featuring strong zincophilicity and rapid diffusion tunnel is selected as the modifying agent to achieve the ion acceleration at interface.

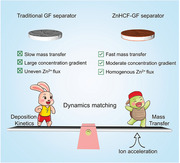

## 1 Introduction

The looming concerns about resource crises and environmental pollution have stimulated the exploitation of renewable energy sources, in which electrochemical energy storage (EES) technology performs a significant role.^[^ ^1^, ^2^, ^3^ ^]^ Owing to high safety, environmental friendliness, and low cost, aqueous zinc‐ion batteries (AZIBs) have been extensively investigated in recent years, showing great potential in large‐scale EES applications.^[^ ^4^, ^5^, ^6^ ^]^ Zinc metal anode has been considered a paramount component for AZIBs due to the high theoretical capacity (820 mAh g^−1^ or 5855 mAh cm^−3^), abundant reserves, low redox potential (−0.76 V vs standard hydrogen electrode), and excellent compatibility in aqueous electrolyte.^[^ ^7^, ^8^, ^9^ ^]^ However, the implementation of the Zn metal anode is hindered by the unsatisfying cycling stability owing to the uncontrolled dendrite growth that would puncture the separator failing batteries.

The issue will be worse when operated at high‐rate conditions due to the mismatch between rapid electrochemical deposition kinetics and slow mass transfer process on the interface of the Zn plate.^[^ ^10^, ^11^, ^12^ ^]^ Specifically, the mismatch will lead to the sluggish ion transport not immediately replenishing the consumed Zn^2+^, giving rise to a surge increase of ion concentration gradient at the near surface of the electrode. Simultaneously, the Zn^2+^ flux will tend to concentrate on a few sites with low nucleation energy, e.g., the tip region, at such a large concentration gradient, aggravating the growth of Zn dendrite.^[^ ^13^, ^14^ ^]^ This problem severely impedes the development of Zn metal anode, especially in high‐rate energy storage devices. Recently, some efforts have been devoted to regulating the kinetics match between faradaic reaction and mass transfer. For example, Zhi and coworkers introduce a steric hindrance effect to slow the electrochemical kinetics to effectively restrain the preferential Zn^2+^ deposition on the tip region.^[^ ^15^ ^]^ However, although dendrite growth can be inhibited, slow total reaction kinetics and large polarization overpotential cannot satisfy the requirement to operate in high‐rate conditions for AZIBs. Accordingly, accelerating ion transport on the electrode surface to modulate the homogenous Zn^2+^ flux is crucial for achieving long‐term stable Zn deposition at high current density.^[^ ^16^ ^]^

The modification for the separator is a convenient strategy to tune the transport behavior of Zn^2+^ because it not only supplies an electron insulative and ion conductive media, but also participates in constructing the interface by directly contacting with electrode. Hitherto, glass fiber (GF) membranes have become the mainstream separator of AZIBs due to their high porosity structure, high ion transmittance, and fantastic wettability with aqueous electrolytes.^[^ ^17^, ^18^, ^19^ ^]^ However, the kinetics mismatch and uneven ion flux at the interface between GF film and Zn anode will uncontrollably emerge and evolve into rapid growth of Zn dendrites, severely downgrading the cycling stability of the system.^[^ ^20^ ^]^ In this regard, the surface decoration on the GF separator is expected to mitigate the dilemma of dendrite growth. For instance, Song et al. constructed a metal‐organic framework functional separator to induce the dominant deposition of the Zn (002) plane, which effectively inhibited the growth of dendrites.^[^ ^21^ ^]^ However, although various modifying agents and corresponding methods for separators have been exploited, the fatal restriction of uneven Zn^2+^ flux at the great concentration gradient has remained unsettled because the speed of ion transport on the interface has not improved.

Herein, the ZnHCF nanoparticle with strong zincophilicity and rapid diffusion tunnel is introduced onto the glass fiber separator by a simple physical method. The customized ZnHCF interface can construct an ion acceleration layer by rapidly trapping Zn^2+^ on the near‐surface and immediately delivering ions onto deposition sites, which can mitigate concentration gradient and modulate homogenize Zn ion flux, achieving long‐term stable Zn^2+^ plating/stripping at high current densities. As a result, the ZnHCF‐modified separator offers Zn||Zn symmetric cells with durable cycle stability of over 2700 h at 2 mA cm^−2^, and an impressive lifespan of 1770 h at a high current density of 10 mA cm^−2^. In addition, the modified separator coupled with different cathode materials, including vanadium oxides, and Prussian blue analogues, could substantially promote the cycling stability and rate capability of the full cells, confirming its great practical feasibility.

## 2 Results and Discussion

**Figure 1:**
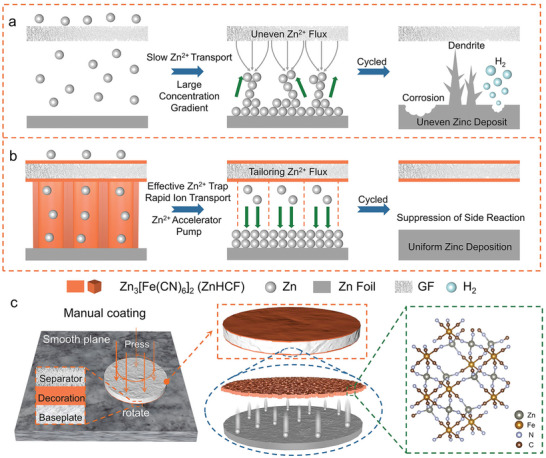
Design of ZnHCF–GF separator. Schematic diagram depicting the Zn^2+^ deposition mechanism using different separators: a) GF, and b) ZnHCF–GF. c) Schematic illustration of the preparation of ZnHCF–GF separator and crystal structure of ZnHCF.

Glass fiber (GF) film is one of the most important separators for AZIBs due to its large porosity and fantastic hydrophilicity. However, the GF separator is unable to ensure long‐term cycling stability of Zn^2+^ plating/stripping because it does not affect the inhibition of dendrite growth and parasitic reactions.^[^ ^22^, ^23^ ^]^ In particular, the high concentration gradient caused by slow ion transport near the Zn surface would induce uneven Zn^2+^ flux in GF to preferentially deposit on the tip area with a low nucleation barrier, aggravating the growth of Zn dendrites (**Figure** **1**a).^[^ ^24^, ^25^ ^]^ The circumstance will worsen at high‐rate conditions owing to the enlarged concentration gradient. To deal with this situation, surface decoration of separators is a simple but effective strategy. Figure 1b presents an ideal surface modification of the GF separator, in which the effective Zn^2+^ capture and fast ion diffusion tunnel for the modified agent could form an ion acceleration layer to modulate Zn^2+^ flux toward more deposition sites, restraining the 2D diffusion concentrated on big nuclei. Thus, the tuned Zn^2+^ flux can guarantee homogeneous dendrite‐free Zn plating. Moreover, parasitic reactions associated with active H_2_O molecules would be also suppressed due to the isolation by the surface modification.

Guided by the thinking of surface decoration for separators, a ZnHCF‐modified GF separator is constructed in this work. As shown in Figure 1c, ZnHCF particles are dispersed and electrostatically adsorbed on the surface of GF by pressing and rotating, in which the facile physical modification is promising in scalable industrial applications. The excellent zincophilicity of ZnHCF could facilitate to rapid Zn^2+^ trap, and the open framework offers a rapid Zn^2+^ diffusion pathway, achieving the ion acceleration from the near surface to deposition sites.^[^ ^26^, ^27^ ^]^ Meanwhile, it can also activate more deposition sites to mitigate flux convergence onto local tip areas. Therefore, the as‐obtained ZnHCF–GF separator is endowed with significant potential in Zn^2+^ flux modulation and robust Zn plating/stripping.

The as‐prepared ZnHCF particle is characterized first. In the X‐ray diffraction (XRD) pattern (Figure S1, Supporting Information), the diffraction peaks of the as‐prepared ZnHCF sample are exactly consistent with those of Zn_3_[Fe(CN)_6_]_2_ (PDF#038–0688).^[^ ^28^ ^]^ All peaks in the Fourier transform infrared (FTIR) spectroscopy (Figure S2, Supporting Information) are attributed to the tensile vibration of the various bonds of ZnHCF.^[^ ^29^ ^]^ Scanning electron microscopy (SEM) and energy dispersive spectrometry (EDS) images (Figure S3, Supporting Information) of ZnHCF intuitively display its nanocube morphology and uniform distribution of elements. The signals of C 1s, N 1s, O 1s, Fe 2p, and Zn 2p are detected in the full X‐ray photoelectron spectroscopy (XPS) spectrum (Figure S4, Supporting Information), and the peak fitting results of Fe 2p and Zn 2p are fully consistent with the valence states of Fe^3+^ and Zn^2+^ in ZnHCF (Figure S5, Supporting Information), which further verifies the successful synthesis of ZnHCF.^[^ ^24^, ^30^ ^]^

**Figure 2:**
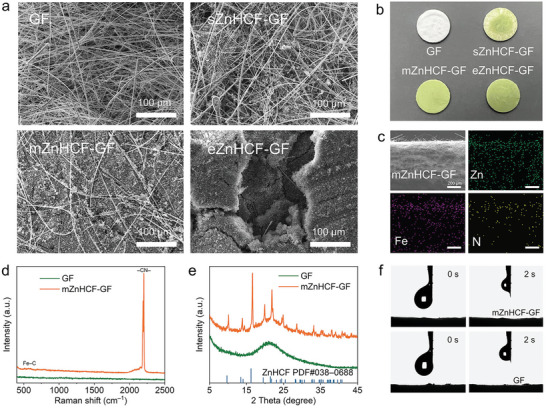
Morphology and structure characterizations of ZnHCF–GF separators. a) SEM images and b) digital photos of GF, sZnHCF–GF, mZnHCF–GF, and eZnHCF–GF separator. c) Cross‐sectional SEM image and corresponding EDS mapping images of mZnHCF–GF film. d) Raman spectra, e) XRD patterns, and f) contact angle of GF and mZnHCF–GF separator, respectively.

To determine the proper dosage of the modifier, the surface morphology for different separators is observed. Compared with bare GF, a small amount of ZnHCF decorated GF (sZnHCF‐GF) presents dispersed particles on the glass fiber, whereas they are not enough to cover the entire separator (**Figure** **2**a; Figure S6, Supporting Information). On the contrary, excessive ZnHCF on GF (eZnHCF‐GF) shows obvious agglomeration and uneven surface on the separator. A moderate amount of ZnHCF powder on GF (mZnHCF‐GF) could maintain a uniform and flat surface, which is expected to serve as an effective modifying layer on the separator. The optical photographs visually display the differences in surface among various separators (Figure 2b). The EDS mappings of the cross‐section for mZnHCF–GF demonstrates that ZnHCF is covered on the GF surface (Figure 2c), which can construct an interface layer with Zn anode to modulate the Zn2+ plating behavior when sealed in the cell.

The modified separator is further characterized to verify its chemical composition. Raman spectra indicate that the characteristic signals for Fe─C and ─CN─ can be well preserved in the mZnHCF–GF separator (Figure 2d).^[^ ^24^ ^]^ Moreover, the peaks in the XRD pattern of mZnHCF–GF are identical with pure ZnHCF (Figure 2e), suggesting that the simple physical treatment for the composite separator induces no change for the ZnHCF modifier. As displayed in Figure 2f, The GF and mZnHCF–GF separator quickly and completely absorb the electrolyte within 2s, indicating that the protective layer of ZnHCF retains the infiltrability of the separator, which is favorable for rapid ion transport on the interface.

**Figure 3:**
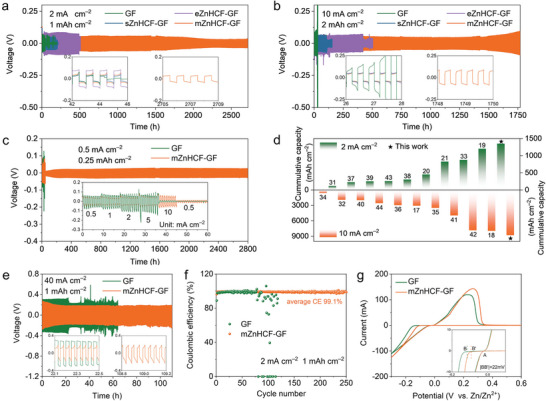
Electrochemical performances and Zn^2+^ deposition behavior with mZnHCF–GF. Cycling performances of Zn||Zn symmetric cells with different separators under different test conditions: a) 2 mA cm^−2^ and 1 mAh cm^−2^; b) 10 mA cm^−2^ and 2 mAh cm^−2^. c) Rate performances of Zn|GF|Zn and Zn|mZnHCF–GF|Zn cells. d) Comparison of Zn||Zn symmetric cells with different separators. e) Cycling performances of Zn|GF|Zn and Zn|mZnHCF–GF|Zn cells at 40 mA cm^−2^ and 1 mAh cm^−2^. f) CE of Zn|GF|Ti and Zn|mZnHCF–GF|Ti cells at a current density of 2.0 mA cm^−2^ with a cut‐off capacity of 1.0 mAh cm^−2^. g) CV curves of Zn|GF|Ti and Zn|mZnHCF–GF|Ti cells at 5mV s^−1^.

The electrochemical behavior of Zn||Zn symmetric batteries assembled with different separators is further investigated. **Figure** **3**a,b compares the electrochemical stability of Zn|GF|Zn and Zn|ZnHCF–GF|Zn cells under two kinds of test conditions. At 2 mA cm^−2^ and 1 mAh cm^−2^, short circuit occurs after ≈45 h for Zn|GF|Zn cell. Remarkably, only the Zn|mZnHCF–GF|Zn cell has the most outstanding stability, which can work continuously for >2700 h with lower overpotential (Figure 3a; Figure S7, Supporting Information). The cycling performance of these symmetric cells is consistent with the experimental expectations obtained from the morphology analysis from SEM images and digital photographs in Figure 2. ZnHCF powder on sZnHCF–GF separator cannot completely cover the GF surface, which leads to the disorder of ion diffusion aperture and uneven distribution of the electric field. Although the eZnHCF–GF separator can be completely covered by a modifier, too thick and uneven layer will lead to an increase in the transport path of Zn ions, which is not conducive to cycle stability, and the high overpotential of Zn|eZnHCF–GF|Zn symmetric cell also verifies this analysis. Therefore, the mZnHCF–GF separator with a uniform and complete protective layer could realize long‐term stable Zn^2+^ plating/stripping. Impressively, the extraordinary endurance for Zn|mZnHCF–GF|Zn symmetric cell is further verified at a high current density of 10 mA cm^−2^, sustaining operation for over 1770 h at a cut‐off capacity of 2 mAh cm^−2^ (Figure 3b; Figure S8, Supporting Information). Moreover, as the current density is increased from 0.5 mA cm^−2^ to 10 mA cm^−2^ with a cut‐off time of 30 min, the Zn|GF|Zn cell is broken down at 10 mA cm^−2^, while the Zn|mZnHCF–GF|Zn cell can still cycle stably for 2800 h after the current density is restored to 0.5 mA cm^−2^ (Figure 3c; Figure S9, Supporting Information), revealing a superior rate performance and cycling durability. Interestingly, the voltage polarization of the Zn|mZnHCF–GF|Zn cell decreases significantly when the current density returns to 0.5 mA cm^−2^, and finally the voltage becomes stable at ≈23 mV, which may be due to the homogeneous Zn^2+^ flux in the ZnHCF layer resulting in an increase in the available nucleation sites for zinc anodes after high current density cycling. Inspiringly, compared with other reported separators, our as‐obtained mZnHCF–GF separator could enable higher cumulative capacity and longer cycle lifespan in Zn‐based symmetric cells (Figure 3d; Table S1, Supporting Information), demonstrating the superiority in electrochemical performances.^[^ ^17^, ^18^, ^19^, ^20^, ^21^, ^31^, ^32^, ^33^, ^34^, ^35^, ^36^, ^37^, ^38^, ^39^, ^40^, ^41^, ^42^, ^43^, ^44^ ^]^ This might be ascribed to that most researches on separator modification neglect the intrinsic limitation of the mismatch between the electrochemical deposition kinetics and ion transfer speed, leading to inferior performance in high‐rate conditions. In addition, even cycled at an extremely high current density of 40 mA cm^−2^, the mZnHCF–GF separator still achieves a stable Zn plating/stripping for an impressive cumulative plating capacity of 2.2 Ah cm^−2^, confirming its promising feasibility at high‐rate Zn batteries due to the ion acceleration design (Figure 3e).

The electrochemical deposition behavior of Zn^2+^ with different separators is further investigated. The Zn||Ti asymmetric cell with bare GF separator only runs for 75 cycles at 2 mA cm^−2^ and 1 mAh cm^−2^ (Figure 3f), probably caused by uncontrolled dendrite growth under a random ion flux field. On the contrary, when the mZnHCF–GF separator is utilized, the cell could render a high average coulombic efficiency (CE) of 99.1% for >250 cycles. In addition, the initial CE of Zn|mZnHCF–GF|Ti cell (96.41%) is greater than that of Zn|GF|Ti cell (88.95%) due to more deposition sites on electrode originating from zincophilic ZnHCF (Figure S10, Supporting Information). Both the initial nucleation overpotential and voltage gap for the 50th cycle of the Zn|mZnHCF–GF|Ti cell are smaller than those of the Zn|GF|Ti cell (Figures S11 and S12, Supporting Information). In addition, nucleation overpotential can be also determined by the voltage difference between A and B/B' in CV curves (Figure 3g), which indicates a lower nucleation overpotential for Zn|mZnHCF–GF|Ti cell. The high CE and low nucleation overpotential validate the effective modulation for surface zincophilicity and deposition sites of the ZnHCF layer on the Zn anode, which is favorable for regulating interface ion flux and guiding homogenous Zn deposition.

**Figure 4:**
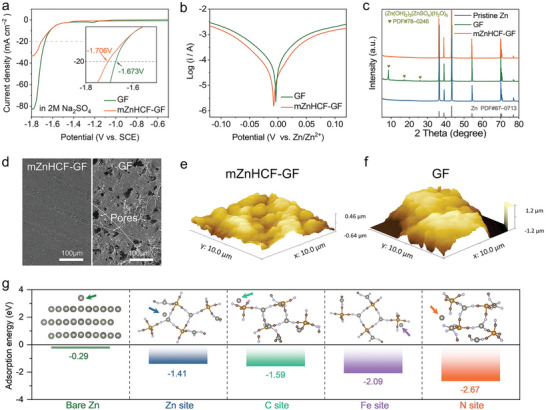
Modulation effect of mZnHCF–GF separator on Zn anode. a) LSV curves of different separators at 5mV s^−1^. b) Tafel curves of Zn|GF|Zn and Zn|mZnHCF–GF|Zn cells. c) XRD patterns, d) SEM images, and e‐f) atomic force microscopy (AFM) images of Zn anodes after 50 plating/stripping at 2 mA cm^−2^ and 1 mAh cm^−2^. g) Adsorption energy and model of Zn^2+^ on the ZnHCF (116) plane of different positions and Zn (002) plane.

The mZnHCF–GF separator also can inhibit side reactions to stabilize the Zn anode by isolating the zinc anode from the electrolyte. The linear sweep voltammetry (LSV) tests are carried out under a three‐electrode system with different separators in 2M Na_2_SO_4_ electrolyte using Zn foil as the working electrode, Ti foil as the counter electrode, and saturated calomel as the reference electrode.^[^ ^45^, ^46^ ^]^ As seen in **Figure** **4**a, for a current density of 20 mA cm^−2^ for hydrogen evolution reaction (HER), the mZnHCF–GF separator could postpone the potential from −1.673 V (vs SCE) to −1.706 V (vs SCE), substantially increasing the difficulty of HER. Tafel plots display that the corrosion current is lowest, and the corrosion resistance is best when the zinc anode is coupled with the mZnHCF–GF separator (Figure 4b). Meanwhile, XRD patterns for Zn electrodes after 50 cycles under 2 mA cm^−2^ and 1 mAh cm^−2^ are collected (Figure 4c), which present an obvious peak of zinc basic salt of (Zn(OH)_2_)_3_(ZnSO_4_)(H_2_O)_5_ (ZSOH, PDF#78–0246) for bare GF based cell.^[^ ^47^ ^]^ In contrast, the by‐product can be inhibited effectively at mZnHCF based system, which can be attributed to the isolation of the Zn anode from the electrolyte by the modifying layer to suppress the parasitic reactions.

Subsequently, the surface morphology for post‐cycling Zn electrodes is observed. As shown in Figure 4d and Figure S13 (Supporting Information), random Zn deposition, a mass of corrosion pores, and byproducts are present on the surface of the Zn plate cycled with bare GF separator. In contrast, a smooth and dense surface with few corrosion pits and byproducts can be obtained for mZnHCF based system. In addition, its flat surface with small height difference is further verified by atomic force microscope (AFM), which is substantially lower than the Zn electrode cycled with GF separator (Figure 4e,f). The flat and dense surface substantiates the effectiveness of the mZnHCF separator in guiding homogenous and dendrite‐free Zn deposition.

Density functional theory (DFT) calculations are employed to investigate the interaction of ZnHCF layer with Zn^2+^.^[^ ^48^ ^]^ As seen in Figure 4g, the adsorption energy of Zn^2+^ on any site of ZnHCF is smaller than that on bare Zn (−0.29 eV), and the lowest adsorption energy on the N site is −2.67 eV, which means that ZnHCF is capable to construct a super zincophilic interface with sufficient nucleation sites to guide the uniform deposition with rapid kinetics. The result is in good agreement with the analysis of the chronoamperometry (CA) test (Figure S14, Supporting Information). Under the implemented potential of −150 mV, the ever‐increasing current trend is present for the Zn plate with bare GF separator, indicating the increase of effective surface area originated from arbitrary 2D diffusion of Zn^2+^.^[^ ^49^ ^]^ Conversely, Zn anode with mZnHCF–GF separator can quickly reach a relatively stable current after initial brief current increase, revealing the inhibition for the 2D diffusion on the Zn plate surface due to the abundant deposition sites and regulated ion transport pathways.

**Figure 5:**
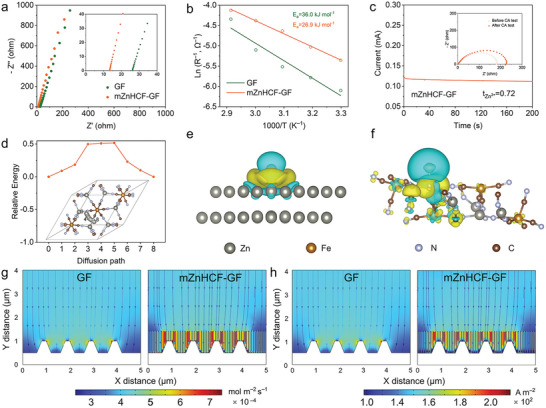
Deposition kinetics and Zn^2+^ flux modulation. a) Nyquist plots and b) activation energy calculation of symmetric cells with different separators. c) CA test of Zn|mZnHCF–GF|Zn cell. Inset: Corresponding Nyquist plots before and after the CA test. d) The diffusion path of Zn^2+^ in ZnHCF and the corresponding DFT calculation of the diffusion energy barrier. Differential charge densities of Zn atom between the e) Zn (002) plane and f) ZnHCF (116) plane, where yellow and cyan correspond to charge accumulation and loss. COMSOL simulations of g) Zn^2+^ flux and h) current density of Zn electrode with GF and mZnHCF–GF separator.

The deposition kinetics of Zn^2+^ through different separators are investigated. It is known that the mass transfer rate of Zn ions lags far behind the electrochemical reaction kinetics in undisturbed system, which will aggravate the uneven deposition of Zn^2+^ at the intense concentration gradient on the electrode surface.^[^ ^50^, ^51^ ^]^ In this sense, it is crucial to accelerate ion transport on the interface to facilitate homogenous Zn deposition. Through the electrochemical impedance spectra (EIS) test, it is found that mZnHCF–GF separator can promote the ion transport within the cell, ion conductivity (σ) of which is determined to be two times higher than that of traditional GF separator (**Figure** **5**a; Figure S15, Supporting Information), and it is also superior to some single‐ion channel research efforts.^[^ ^24^ ^]^ This is fully consistent with the DFT results, indicating that, the strong interaction between ZnHCF and Zn^2+^ causes Zn^2+^ to move rapidly through the diffusion layer to match the kinetics between ion transport and charge transfer. To further evaluate the influence of reaction kinetics of Zn deposition with different separators, the activation energy (*E_a_*) is calculated according to the Arrhenius equation by testing EIS under different temperatures (Figure S16, Supporting Information).^[^ ^52^, ^53^ ^]^ As seen in Figure 5b the *E_a_* for mZnHCF–GF separator system (26.9 kJ mol^−1^) is smaller than that of the GF separator (36.0 kJ mol^−1^). According to the data of CA and EIS test in Figure 5c and Figure S17 (Supporting Information), the Zn^2+^ transference number ($t_{Zn^{2+}}$) for Zn|mZnHCF–GF|Zn cell is calculated as 0.72, which is substantially higher than that of bare GF of 0.46.^[^ ^54^, ^55^ ^]^ The results demonstrate that the rapid capture of Zn^2+^ from the diffusion layer by the ZnHCF layer alleviates the concentration gradient between the diffusion layer and the Helmholtz layer and achieves surface ion acceleration, which regulates the ion flux and inhibits the growth of Zn dendrites (Figure S18, Supporting Information).

To comprehensively understand the ion acceleration behavior with the mZnHCF–GF separator, DFT calculations are conducted to reveal the transport process on the interface. A low diffusion energy barrier of 0.51 eV within the ZnHCF is determined (Figure 5d), indicating that Zn^2+^ can readily migrate the ZnHCF layer, which has a large number of pathways to supply sufficient diffusion efficiency. Importantly, the more electron transference for the differential charge density between Zn^2+^ and ZnHCF crystal plane reveals a stronger interaction than that with Zn plate (Figure 5e,f; Figures S19 and S20, Supporting Information), which is consistent with the results of adsorption energy calculated before. Therefore, the zincophilic ZnHCF rapidly captures Zn^2+^ at the Zn anode interface and deliver it to deposition site immediately, thereby accelerating the ion transport at the interface to moderate the ion concentration gradient and ensure uniform and long‐lasting·Zn deposition.

The manipulation for Zn^2+^ flux upon the surface of Zn metal anode with mZnHCF–GF separator is further investigated by finite element modeling (FEM) simulations. As seen in Figure 5g, Zn^2+^ flux tends to concentrate on the tip region of the Zn anode to form the tip effect, which would lead to serious dendrite growth. In contrast, the redistribution of Zn^2+^ flux can be achieved with the implementation of mZnHCF‐GF separator. Owing to the ion acceleration effect of the introduced ZnHCF interface on the Zn surface, more Zn^2+^ flux can be transported to the smooth area of the waist and base, rather than the mere apical part of the Zn hump, facilitating homogenous Zn deposition. The simulated ion flux distribution plot exhibits higher total ion flux for mZnHCF‐GF, as well as denser deposition sites, than the free GF separator system (Figure S21, Supporting Information). In addition, the distribution for simulated surface current density is in good agreement with the results for ion flux (Figure 5h; Figure S22, Supporting Information), demonstrating the effectiveness of the ZnHCF modifying layer on stabilizing Zn deposition and promoting ion transport kinetics.

**Figure 6:**
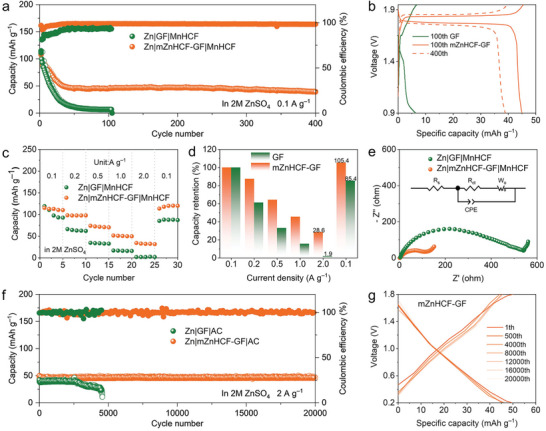
Electrochemical performances of full cells. a) Cycling performances and b) charge/discharge curves of Zn|GF|MnHCF and Zn|mZnHCF–GF|MnHCF batteries at 0.1 A g^−1^. c) Rate performances, d) corresponding capacity retention, and e) Nyquist plot for Zn||MnHCF cells based on different separators. f) Cycling performances and g) charge/discharge curves of Zn|GF|AC and Zn|mZnHCF–GF|AC capacitors at 2.0 A g^−1^.

To validate the feasibility of the mZnHCF–GF separators in practice, manganese hexacyanoferrate (MnHCF) and activated carbon (AC) as cathode materials are used for assembling zinc‐ion battery and zinc‐ion capacitor, respectively. The morphology and structure of as‐prepared MnHCF are available in Figures S23 and S24 (Supporting Information).^[^ ^30^, ^56^ ^]^ As seen in **Figure** **6**a, the cell with bare GF separator shows rapid capacity decline and eventually breaks down after 110 cycles, while mZnHCF–GF endows the cell with superior cycling stability for >400 cycles. Moreover, the high voltage plateau of MnHCF can be well reserved in mZnHCF–GF system (Figure 6b), meaning the suppression for decomposition of the PBA cathode. To clarify the mere role of interface modification for the ZnHCF layer on GF instead of supplying extra capacity or voltage, a Zn|mZnHCF–GF|Ti cell without applying cathode material is assembled (Figure S25, Supporting Information), which exhibits few effective discharge capacities. Moreover, it can be observed through SEM images that the ZnHCF on the modified separator is not dislodged during the cycling process (Figure S26, Supporting Information). In addition, the rate capability for the Zn||MnHCF battery can be substantially improved with mZnHCF–GF separator (Figure 6c,d; Figure S27 and Table S2, Supporting Information), attributed to the ion acceleration effect of the ZnHCF interfacial layer. Meanwhile, the Zn|mZnHCF–GF|MnHCF battery has a lower charge transfer resistance (R_CT_) in comparison with the Zn|GF|MnHCF cell (Figure 6e; Table S3, Supporting Information), which confirms that mZnHCF separator can promote the kinetics of interfacial charge transfer. The long‐term reliability at high‐rate condition of the mZnHCF–GF separator is further validated in Zn||AC capacitor system. As shown in Figure 6f,g, the capacitor with bare GF separator exhibits remarkable capacity attenuation after 2700 cycles at 2 A g^−1^ and rapidly fails at ≈4800 cycles, while Zn|mZnHCF–GF|AC capacitor can stably operate over 20 000 cycles with high capacity retention of 95.8%. Moreover, Zn|mZnHCF–GF|AC capacitor features superior rate performance at different current densities up to 20 A g^−1^ in comparison with bare separator (Figure S28, Supporting Information). The results substantiate the significant superiority in prolonged cycling stability and promoted interface reaction kinetics of mZnHCF–GF separators in practical application.

## 3 Conclusion

In summary, a facile separator engineering has been developed to tailor ion flux and optimize the reaction kinetics on the surface of the Zn anode, achieving long‐last and dendrite‐free Zn deposition. The ZnHCF modifying layer can moderate the concentration gradient on the surface of the Zn plate by ion acceleration effect and modulate Zn^2+^ flux to enable homogenous deposition. Consequently, the mZnHCF–GF separator endows Zn||Zn symmetric cell with a long lifetime of >2700 h at 2 mA cm^−2^ and an impressive cyclability of 1770 h at 10 mA cm^−2^, which surpasses most modified separators previously reported. In addition, when coupled with different cathode materials to construct full cells, the mZnHCF–GF‐based systems exhibit superior cycling stability and rate performance in comparison with bare separators, validating its good promise in practical applications of AZIBs. This work highlights the crucial role of accelerating ion transport and regulating Zn^2+^ flux of near‐surface regions in developing long‐lasting and high‐rate Zn metal batteries.

## 4 Experimental Section

### Preparation of ZnHCF

ZnHCF was synthesized by co‐precipitation at room temperature. Specifically, solution A was formed by dissolving 0.005 mol ZnSO_4_ in 50 mL deionized water. Solution B was formed by dissolving 0.005 mol K_3_Fe(CN)_6_ in 100 mL deionized water (DIW). Solution A was slowly dropwise added to solution B by a peristaltic pump. The mixture was aged at room temperature for 24 h. Then, the precipitation was centrifuged several times with ethanol and DIW. Finally, it was dried overnight in a vacuum environment of 60 °C.

### Preparation of MnHCF

The steps for the synthesis of MnHCF are almost identical to those for ZnHCF, except for the adjustment of solutions A and B. Specifically, solution A was formed by dissolving 0.0005mol MnSO_4_ and 1g SC in 50 mL DIW. Solution B was formed by dissolving 0.001mol K_3_Fe(CN)_6_ in 50 mL DIW.

### Preparation of ZnHCF—GF

First, a certain amount of ZnHCF powder was scattered in the mortar (or other smooth surface). Then, by pressing a glass fiber separator (Whatman GF/D) with a diameter of 1.6cm on ZnHCF and sliding quickly, ZnHCF was uniformly attached to the GF using electrostatic adsorption and friction. The same process was performed on the other side of the GF to obtain ZnHCF–GF. In the text, 3, 5, and 7 mg ZnHCF powder were used in sZnHCF–GF, mZnHCF–GF, and eZnHCF—GF, respectively. The detailed preparation methods can be available in Movie S1 (Supporting Information).

### Battery Assembling

At room temperature, half/full batteries (Zn|GF|Zn, Zn|ZnHCF–GF|Zn, Zn|GF|Ti, Zn|ZnHCF–GF|Ti, Zn|GF|AC, Zn|ZnHCF–GF|AC, Zn|GF|MnHCF, Zn|ZnHCF–GF|MnHCF) of the different separators are assembled in CR–2032 coin–type batteries. All of these processes use polished Zn plates (thickness 50µm, ϕ = 16mm), 2M ZnSO_4_ electrolyte, and GF/ZnHCF–GF separator. The cathode (ϕ = 12mm) was coated on a Ti foil (thickness 20µm) after mixing the active material (MnHCF/AC), conductive material (acetylene black), and adhesive (polyvinylidene fluoride) in a mass ratio of 7:2:1, and then dried in a vacuum oven at 80 °C for 12 h. The mass loading density of the electrode was ≈2 mg cm^2^.

## Conflict of Interest

The authors declare no conflict of interest.

## Supporting information



Supporting Information

Supplemental Movie 1

## Data Availability

The data that support the findings of this study are available from the corresponding author upon reasonable request.
