# Supplementary material for: Tailoring Zn2+ Flux by an Ion Acceleration Layer Modified Separator for High‐Rate Long‐Lasting Zn Metal Anodes
Source: Adv Sci (Weinh). 2024 Oct 8;11(44):2407410. doi: 10.1002/advs.202407410 (PMC11600266; doi:10.1002/advs.202407410)
Supplement: Supplementary file 1 — Supporting Information [file ADVS-11-2407410-s001.docx]

Supporting Information

Tailoring Zn^2+^ Flux by an Ion Acceleration Layer Modified Separator for High-Rate Long-Lasting Zn Metal Anodes

Yicheng Tan, Duo Chen^*^, Tengyu Yao, Yiming Zhang, Chenglin Miao, Hang Yang, Yuanhang Wang, Li Li, Volodymyr Kotsiubynskyi, Wei Han, and Laifa Shen^*^

**Materials**

Zinc sulfate heptahydrate (ZnSO_4_·7H_2_O, 99.5%), hydrochloric acid (HCl), and hydrogen peroxide (H_2_O_2_, 30%) were produced by Nanjing Chemical Reagent Co., Ltd.

Aniline and sodium citrate (SC) were purchased from Shanghai Macklin Biochemical Co., Ltd. Manganese sulfate monohydrate (MnSO_4_·H_2_O) was purchased from Shanghai Hao Hong Biological Medicine Technology Co., Ltd. Potassium ferricyanide (K_3_Fe(CN)_6_, 99.5%) and activated carbon (AC) powder were supplied by Shanghai Aladdin Biochemical Technology Co., Ltd.

**Electrochemical measurements**

Galvanostatic charge/discharge (GCD) cycling and rate tests of symmetric (Zn|GF|Zn, Zn|ZnHCF–GF|Zn) /full cells (Zn|GF|AC, Zn|ZnHCF–GF|AC, Zn|GF|MnHCF, Zn|ZnHCF–GF|MnHCF) and Coulombic efficiencies tests for asymmetric cells (Zn|GF|Ti, Zn|ZnHCF–GF|Ti) were carried out on the LAND CT2001A battery–testing system. Other electrochemical tests of asymmetric/symmetric cells were performed on a Bio-Logic VSP electrochemical workstation. The specific parameters in the above test are as follows:

The Zn||AC, and Zn||MnHCF batteries were performed in the potential ranges of 0.2–1.8 V and 0.9–1.95 V (vs. Zn^2+^/Zn), respectively. Rate performances of Zn||MnHCF batteries were obtained at current densities of 0.1, 0.2, 0.5, 1.0, 2.0, and 0.1 A g^−1^, rate performances of Zn||AC batteries were obtained at current densities of 0.1, 0.5, 1.0, 2.0, 5.0, 10.0, 20.0 and 0.1 A g^−1^. Cyclic voltammetry (CV) tests of asymmetric cells were conducted at a scan rate of 5 mV s^−1^. Linear polarization curve (LSV) was carried out in 2M NaSO_4_ with three electrodes, in which Zn foil was the working electrode, Ti foil was the counter electrode, and saturated calomel was the reference electrode. Tafel tests of symmetric cells were carried out at a scan rate of 5 mV s^−1^. Chronoamperogram (CA) tests of symmetric cells under a potential of 150 mV and 10 mV (Zn^2+^ transference number test). All tests related to EIS are performed in the frequency range of 0.01 to 100,000 Hz. To calculate the ionic conductivity ($\sigma$), EIS test results of Ti|GF|Ti and Ti|ZnHCF–GF|Ti cells were analyzed and then the $\sigma$ value was calculated according to *Eq*. S1:

$\sigma=\frac{L}{R_{b}S}$ *Eq*. S1

where $L$ is the thickness of separators,$R_{b}$ is the resistance of asymmetric cells, and $S$ is the effective contact area of separators.

To compare the activation energy ($E_{a}$) of different separators, different temperatures EIS tests of symmetric cells were implemented. Then the $E_{a}$ was sorted out by applying *Eq*. S2:

$\frac{1}{R_{ct}}=Aexp(-\frac{E_{a}}{RT})$ *Eq*. S2

where $R_{ct}$ represents the charge transfer resistance of symmetric cells, $R$ represents the gas constant, and $T$ represents different temperatures.

Besides, the Zn^2+^ transference number of symmetric cells with different separators was estimated based on the EIS and CA tests. The formula is as *Eq*. S3:

$t_{{Zn}^{2+}}=\frac{I_{S}(\triangle V-I_{0}R_{0})}{I_{0}(\triangle V-I_{S}R_{S})}$ *Eq*. S3

where $\triangle V$ represents the applied constant polarization potential (10 mV). $I_{0}$ and $I_{S}$ are the initial and steady-state current in the CA tests, respectively. $R_{0}$ and $R_{S}$ are the charge transfer resistance before and after the CA tests, respectively.

**Density functional theory (DFT) calculations**

Based on the plane–wave basis sets, the Vienna ab initio simulation package (VASP) used the projector augmented–wave method^[1-2]^ to calculate the spin-polarized DFT calculations^[3-4]^. The exchange-correlation potential was treated by using a generalized gradient approximation (GGA) with the Perdew-Burke-Ernzerhof (PBE) parametrization^[5]^. To avoid interaction between adjacent images, a vacuum area of about 15 A was used. The energy cutoff was set to be 450 eV. The Brillouin–zone integration was sampled with a Γ-centered Monkhorst-Pack mesh^[6]^ of 2 × 2 × 1 for the Zn (0 0 2) surface and 1 × 1 × 1 for the ZnHCF (1 1 6) surface. The structures were outright relaxed until the maximum force on each atom was less than 0.03 eV/Å, and the energy convergent standard was 10–5 eV.

The correlation different charge density is calculated as follows:

$\Delta\rho=\rho_{s}-\rho_{is}-\rho_{im}$ *Eq*. S4

where $\rho_{s}$, $\rho_{is}$, and $\rho_{im}$ are the electron densities of the molecule adsorbed on the surface, the isolated surface and the isolated molecule, respectively.

The adsorption energy (E_ads_) of Zn at different positions can be calculated as:

$E_{ads}=E_{Zn-sub}-E_{Zn}-E_{sub}$ *Eq*. S5

where $E_{Zn-sub}$ stands for the energy of the surface with the adsorbed molecule, $E_{Zn}$ is the energy of the surface, and $E_{sub}$ is the energy of the isolated molecule. The diffusion energy barrier is obtained by CI-NEB method and seven intermediate states are selected.

**COMSOL finite element analysis**

COMSOL Multiphysics software was used to simulate the current density and Zn^2+^ flux at the electrode-electrolyte interface. In the simulation, the electrode surface reaction obeys the Butler-Volmer expression, and the flux of each ion obeys the Nernst−Planck formulation. The length and width of the 2D cell model are 5 and 4 μm, respectively, and the humps with a height of 0.5 μm represent the Zn grain. The rectangular array (0.02 μm in width) represents the ZnHCF layer with carrier channels. The anode potential is 0V and the initial Zn^2+^ cation concentration is 2M.

**Supporting Figures**


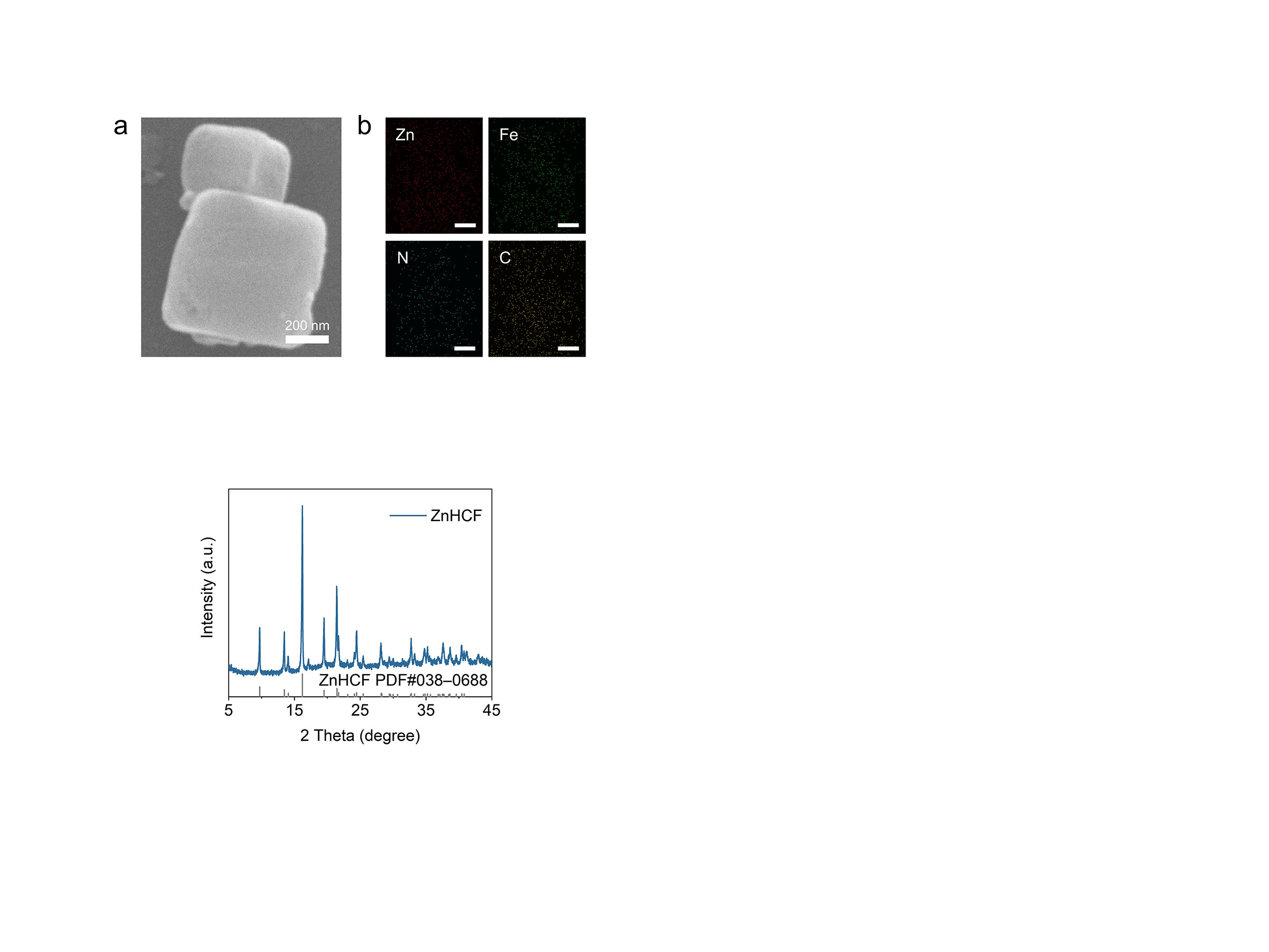


**Figure S1.** XRD pattern of the as-prepared ZnHCF powder.


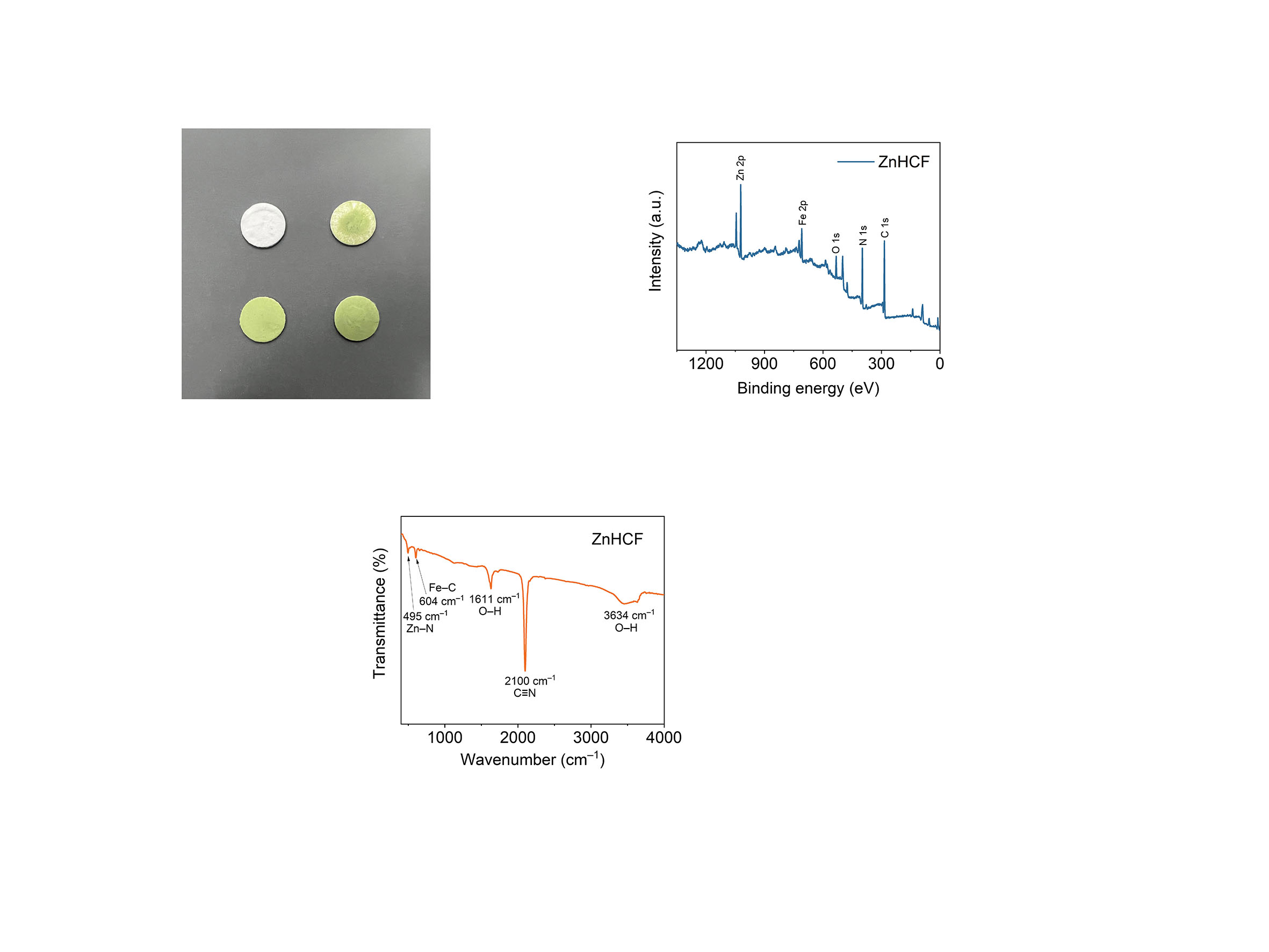


**Figure S2.** FTIR spectra of the as-prepared ZnHCF powder.


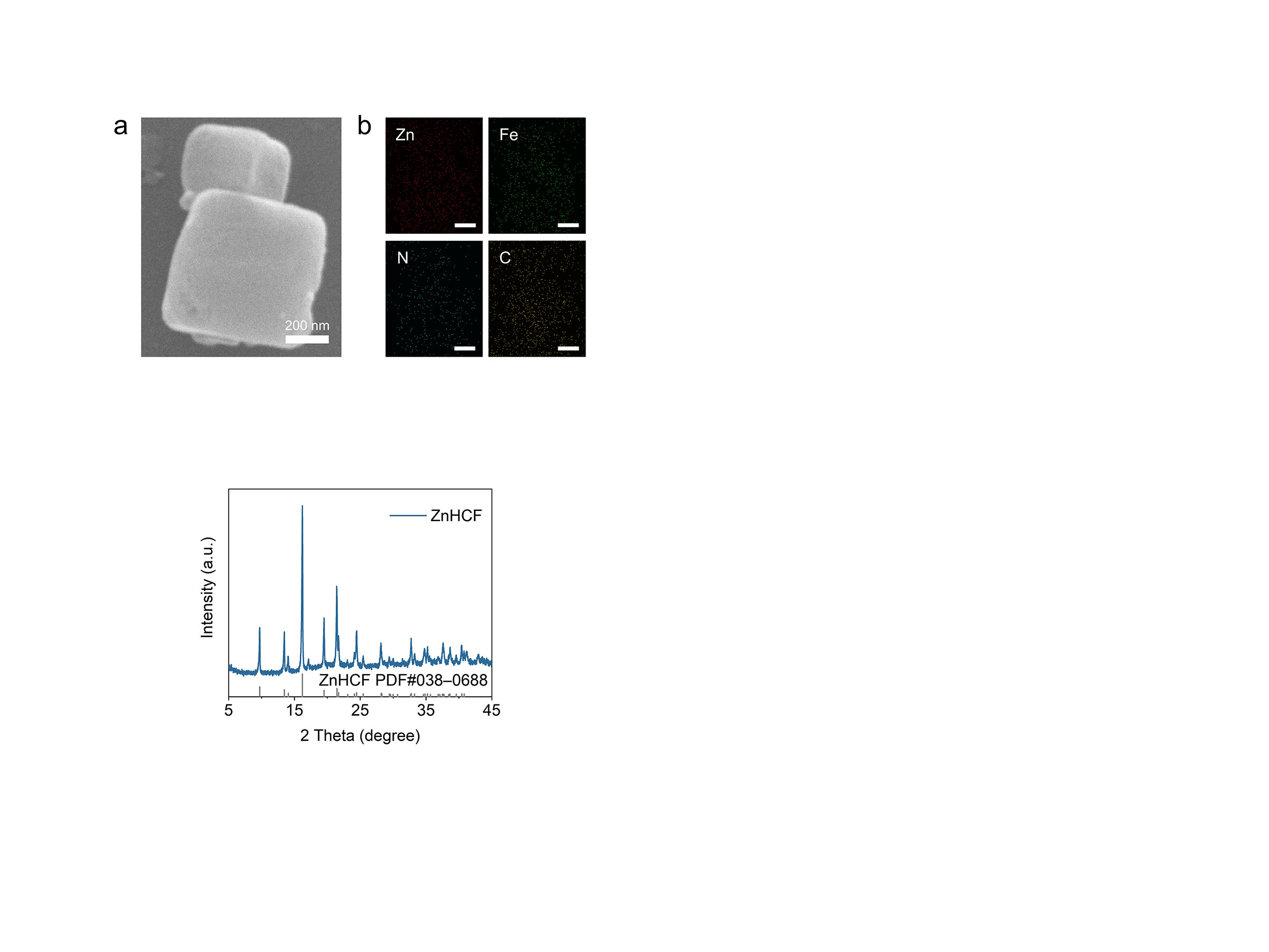


**Figure S3.** (a) SEM images of the as-prepared ZnHCF powder and (b) the corresponding EDS image.


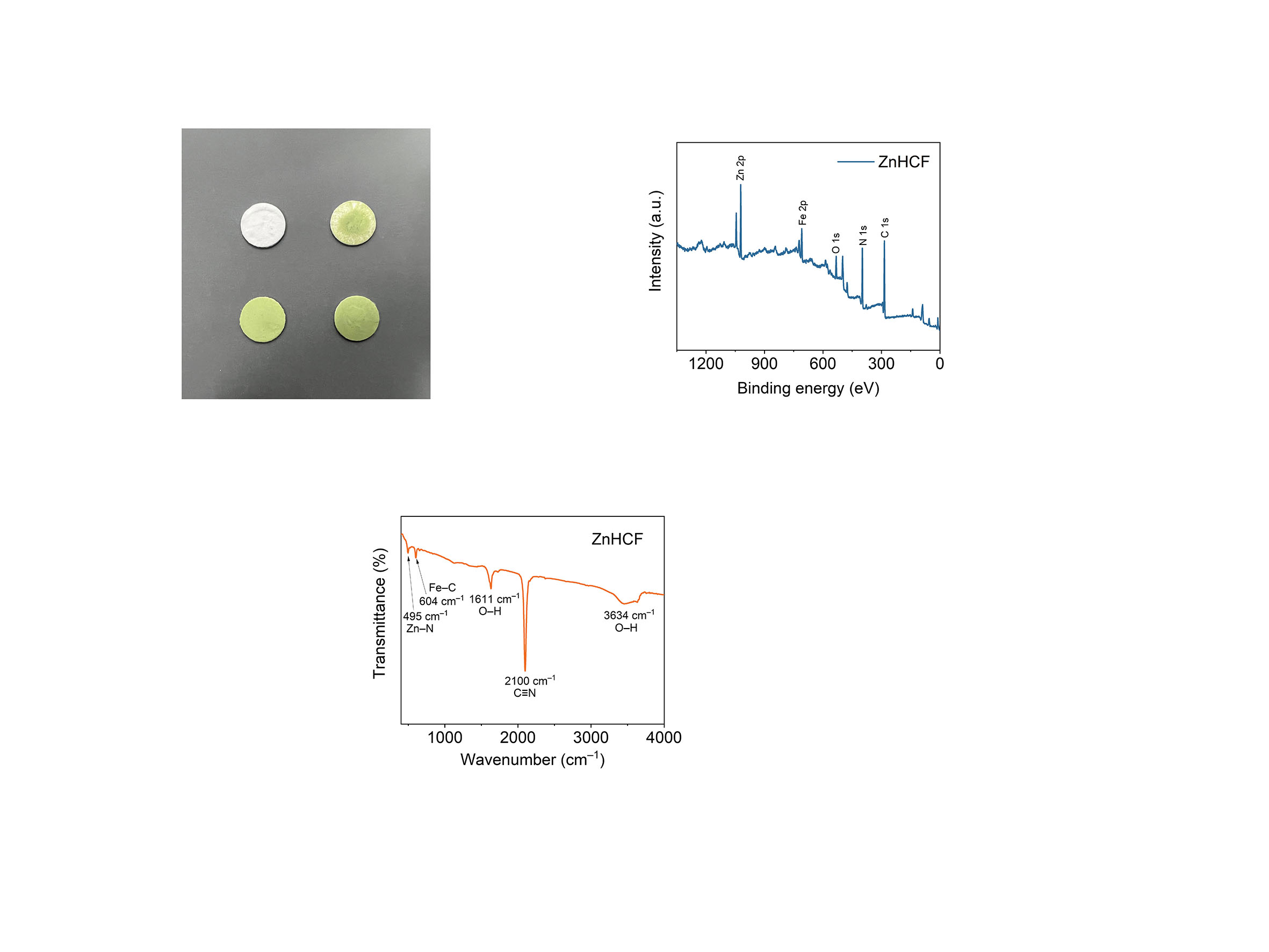


**Figure S4.** XPS full spectrum of the as-prepared ZnHCF powder.


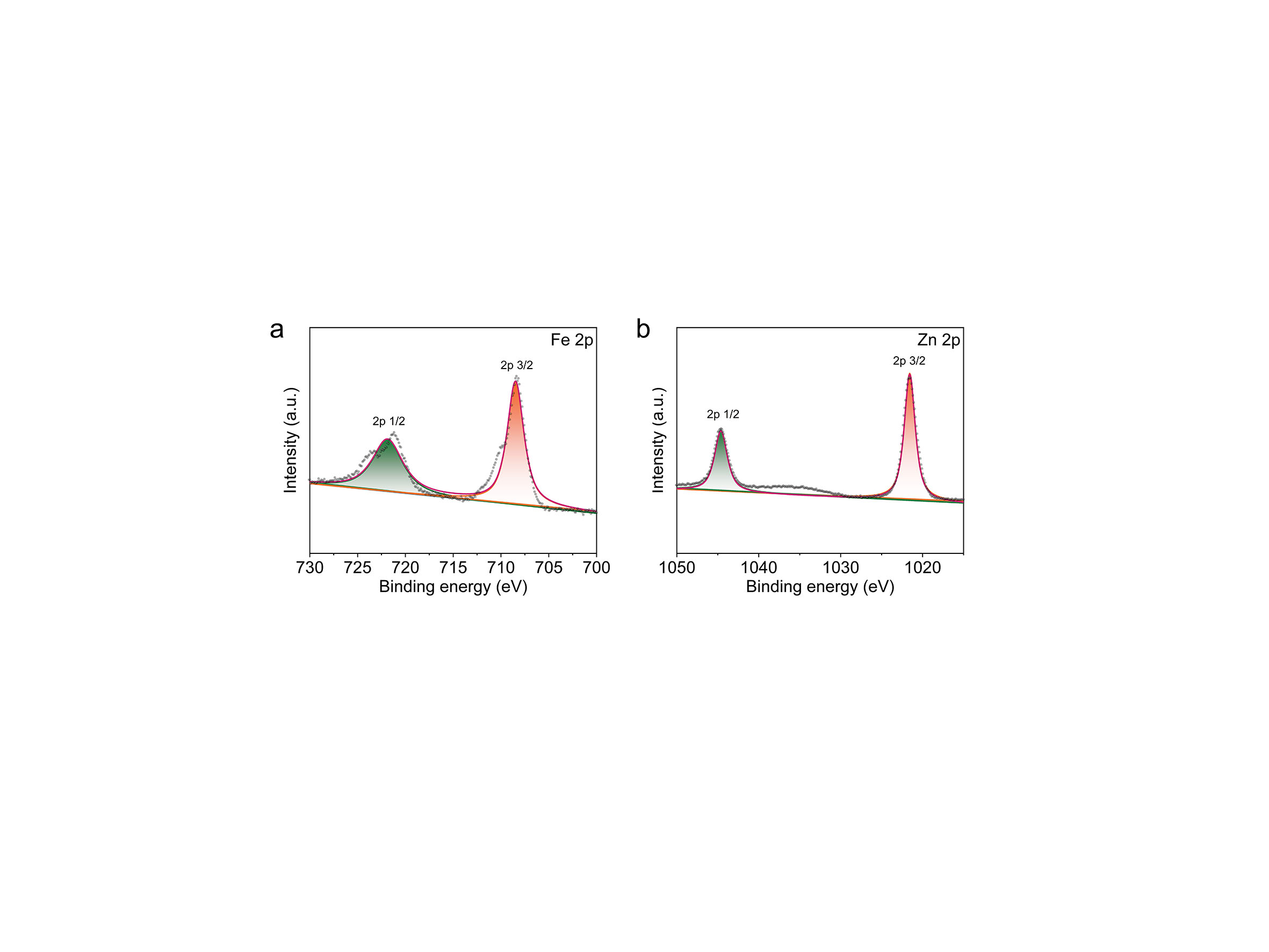


**Figure S5.** XPS spectra of a) Fe 2p and b) Zn 2p for the as-prepared ZnHCF powder.


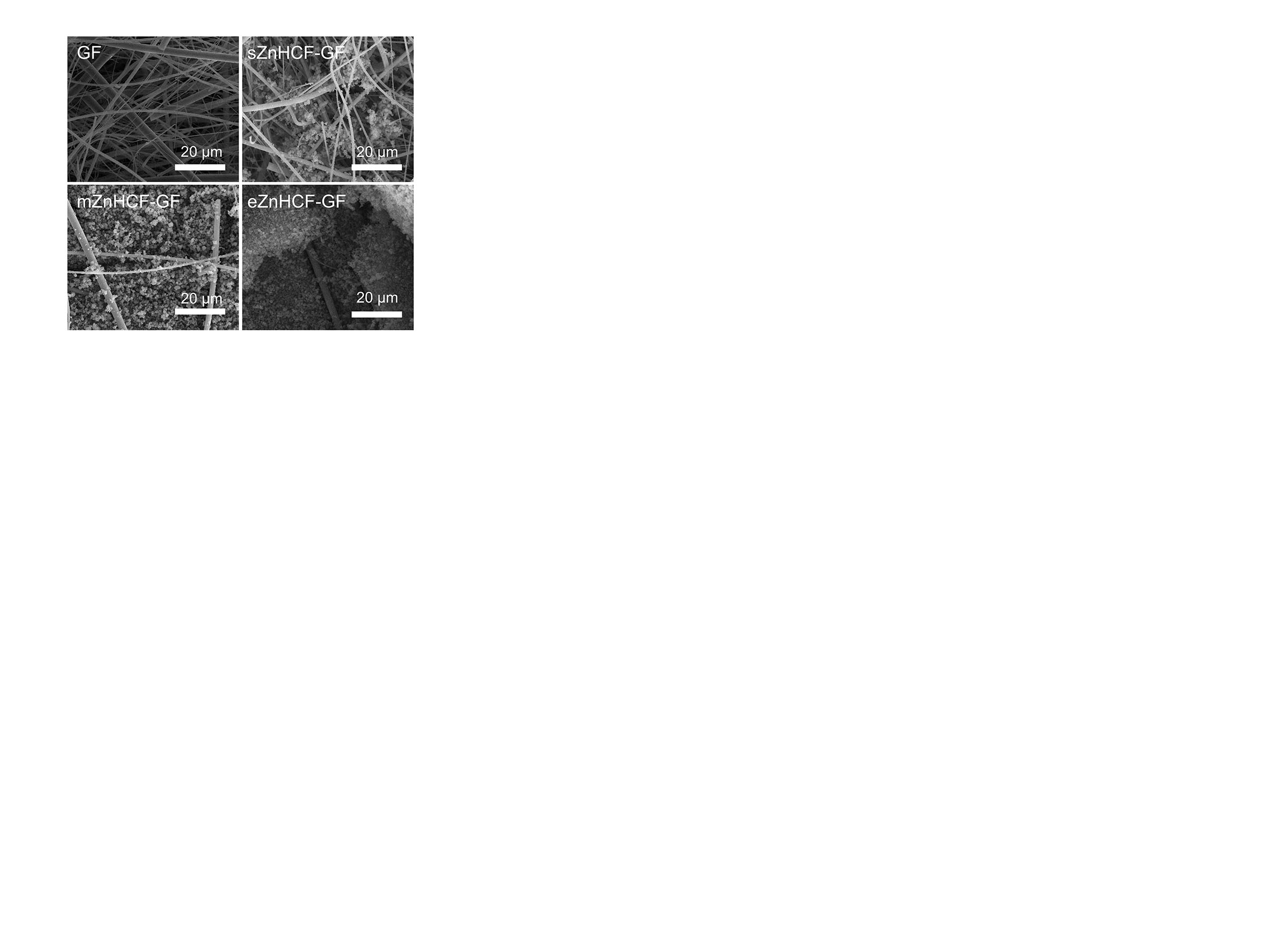


**Figure S6.** SEM images of GF, sZnHCF–GF, mZnHCF–GF, and eZnHCF–GF separator.


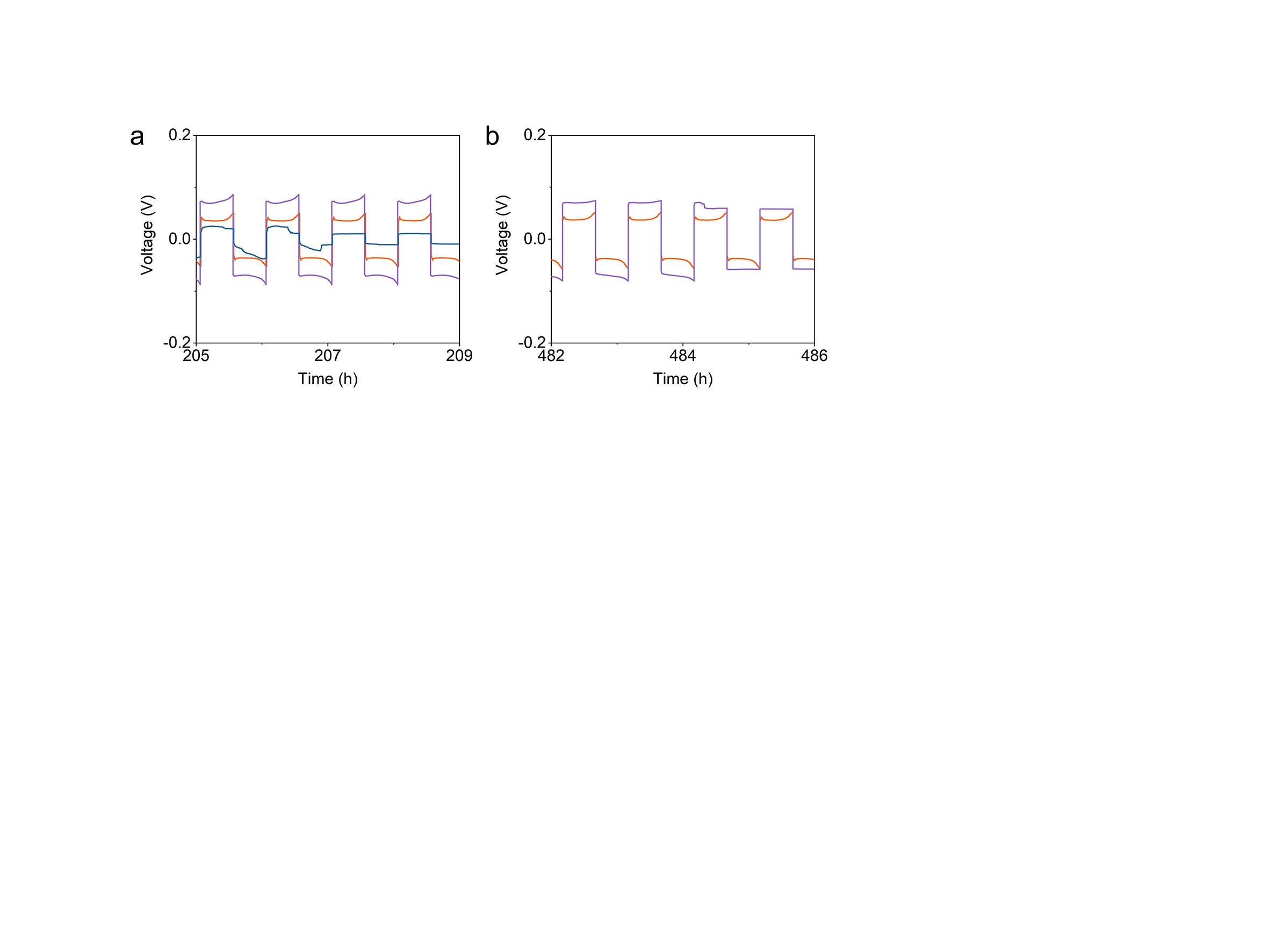


**Figure S7.** The enlarged view of the cyclic performance details of partial symmetrical cell system at 2 mA cm^−2^ and 1 mAh cm^−2^.


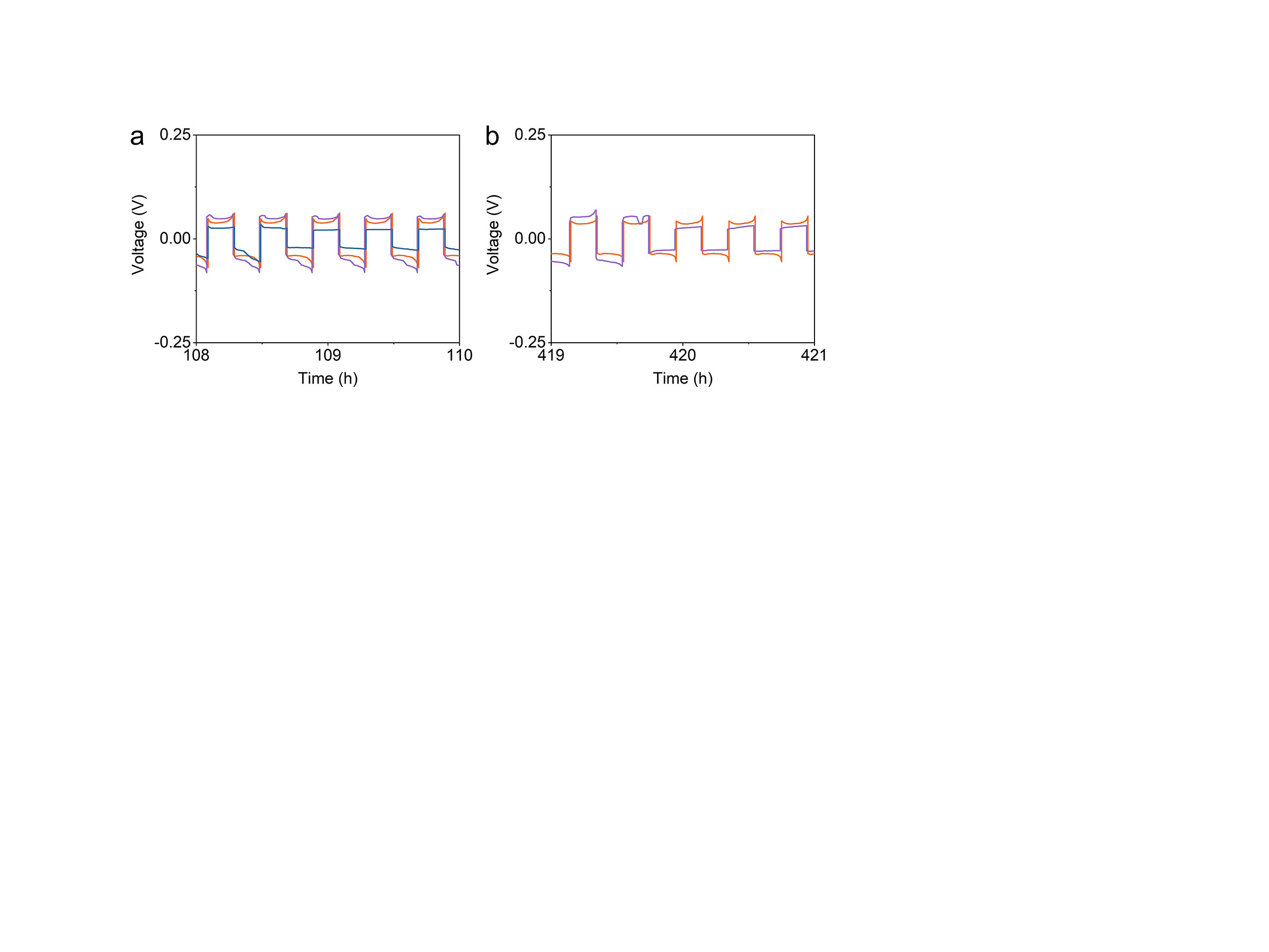


**Figure S8.** The enlarged view of the cyclic performance details of partial symmetrical cell system at 10 mA cm^−2^ and 2 mAh cm^−2^.


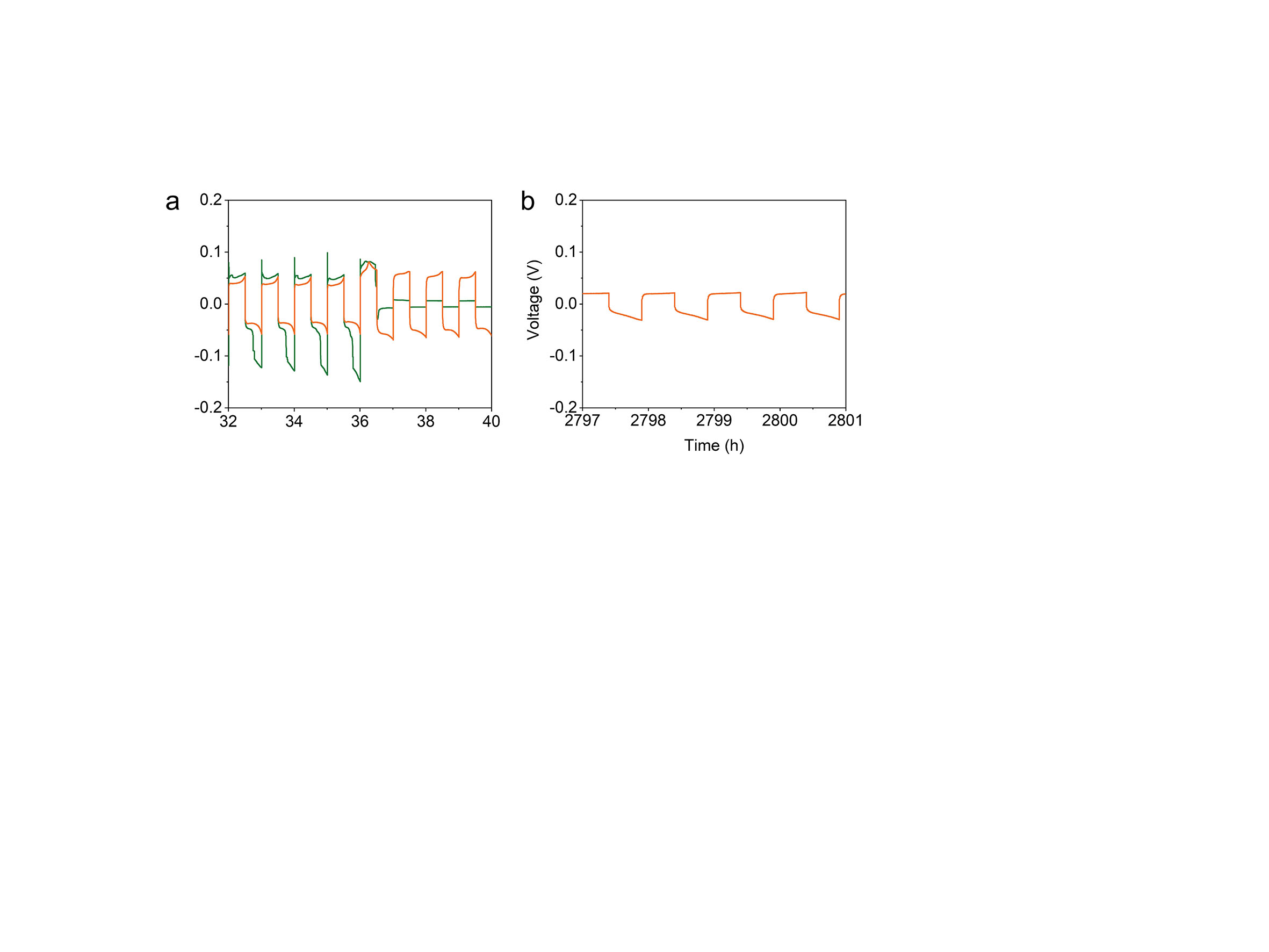


**Figure S9.** The enlarged view of the rate performance details of Zn|mZnHCF–GF|Zn cell.


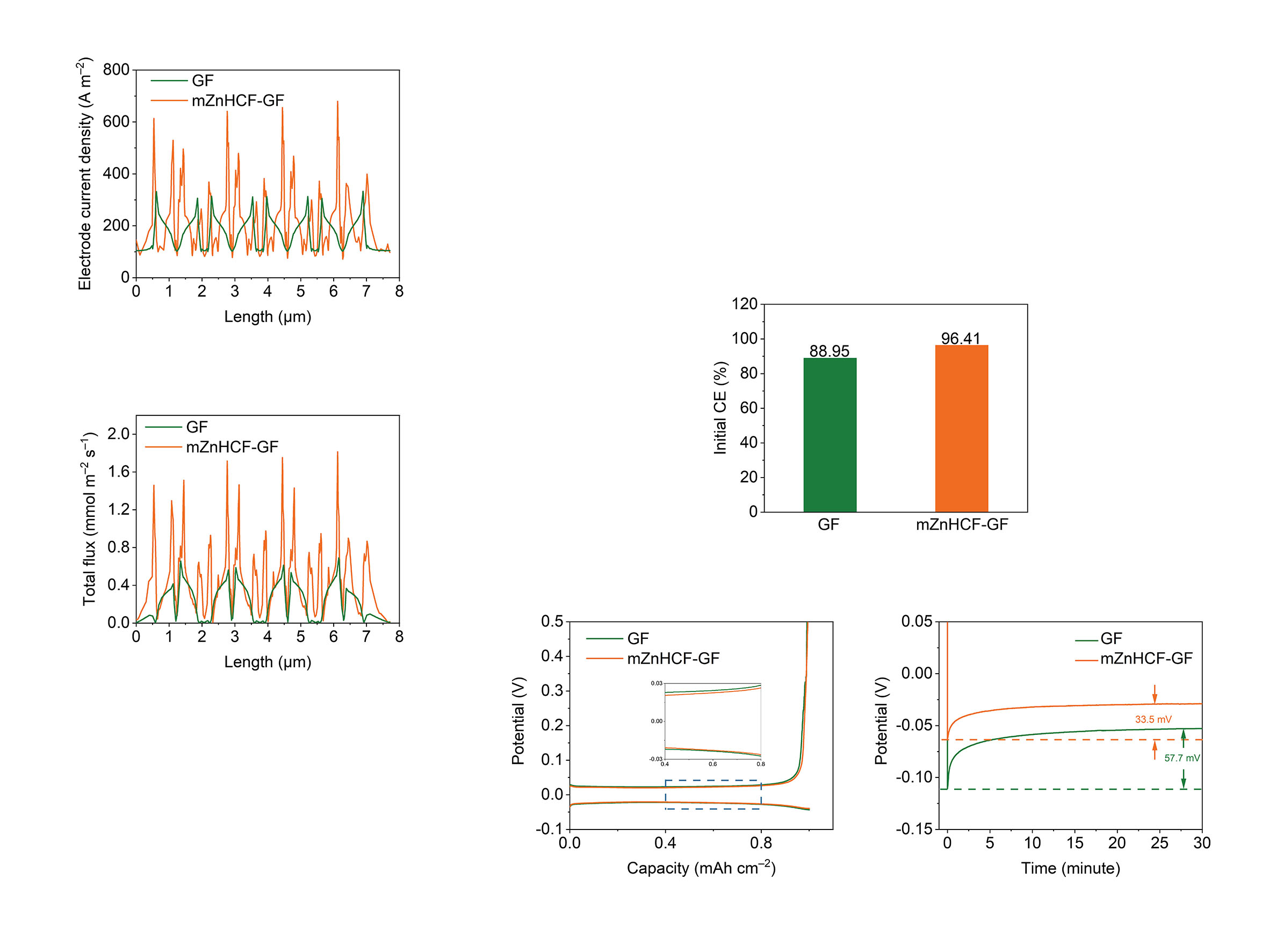


**Figure S10.** Initial CE of Zn|GF|Ti and Zn|mZnHCF–GF|Ti cell.

**
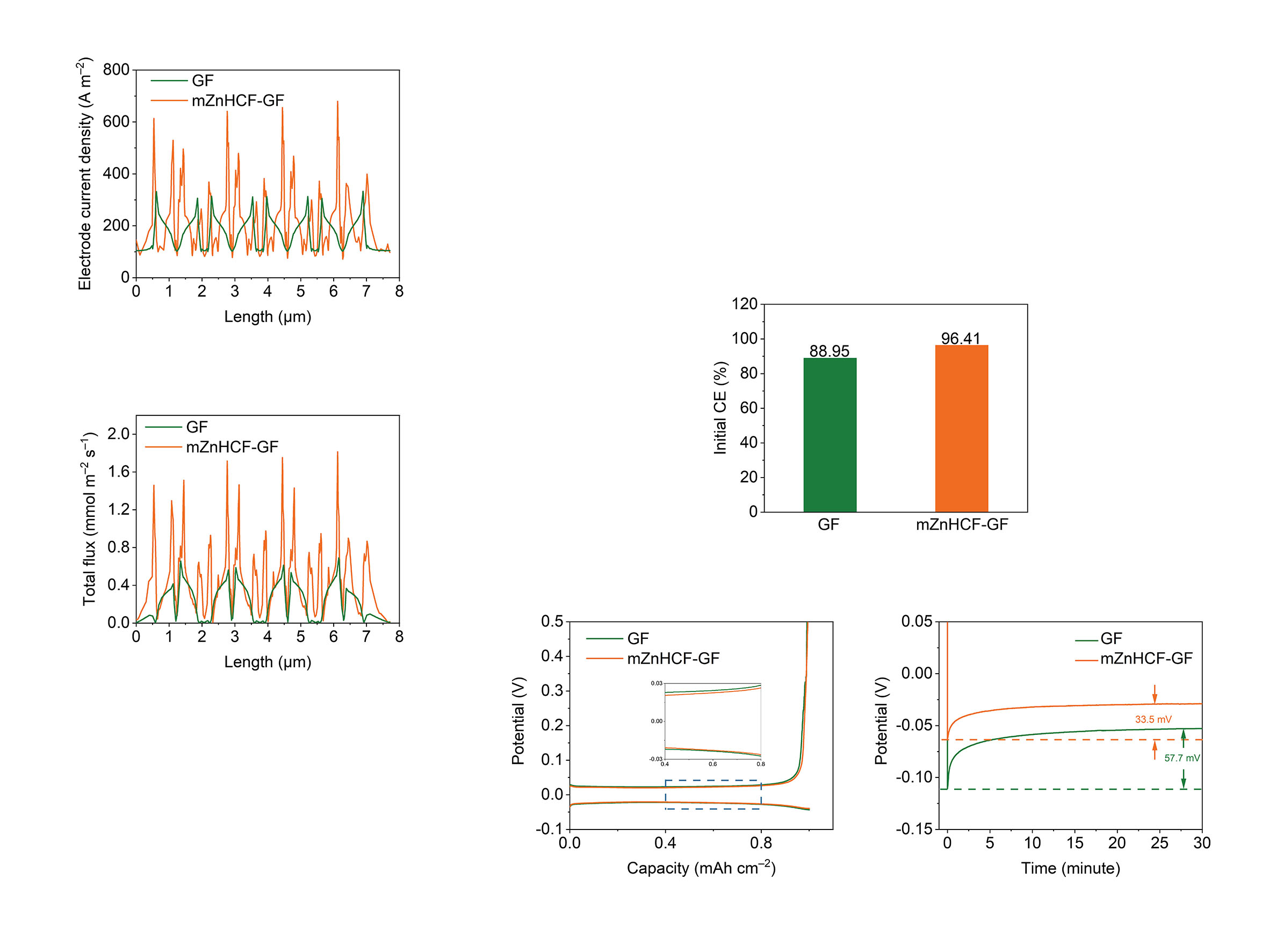
**

**Figure S11.** The nucleation overpotential of Zn|GF|Ti and Zn|mZnHCF–GF|Ti cells at a current density of 2.0 mA cm^−2^ with a cut-off capacity of 1.0 mAh cm^−2^.

**

**

**Figure S12.** The plating/stripping profiles at the 50th cycle of Zn|GF|Ti and Zn|mZnHCF–GF|Ti cells at a current density of 2.0 mA cm^−2^ with a capacity of 1.0 mAh cm^−2^.

**
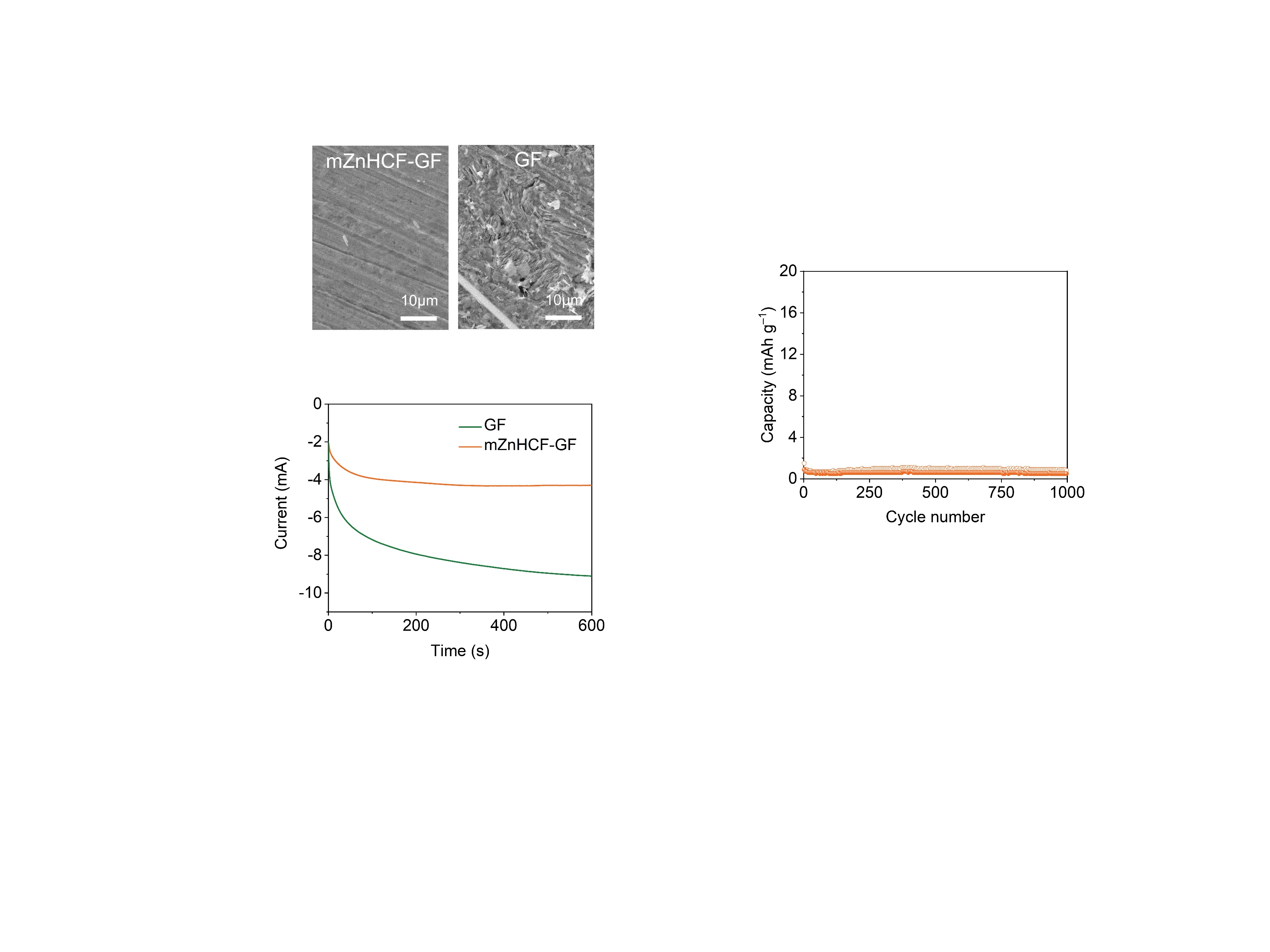
**

**Figure S13.** SEM images of Zn anodes after 50 plating/stripping at 2 mA cm^−2^ and 1 mAh cm^−2^.


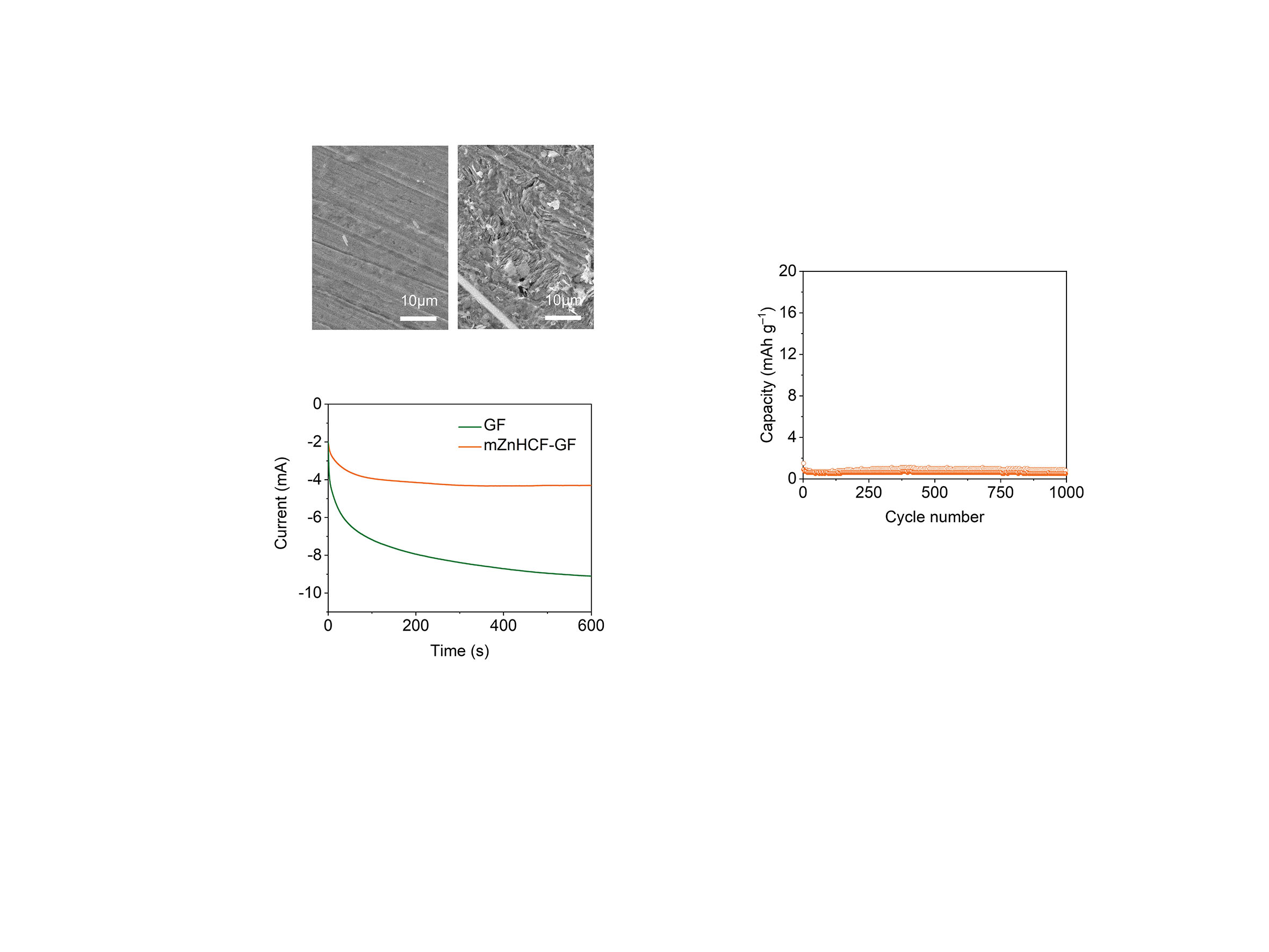


**Figure S14.** CA curves of Zn|GF|Zn and Zn|mZnHCF–GF|Zn cell under an overpotential of −150mV.

**
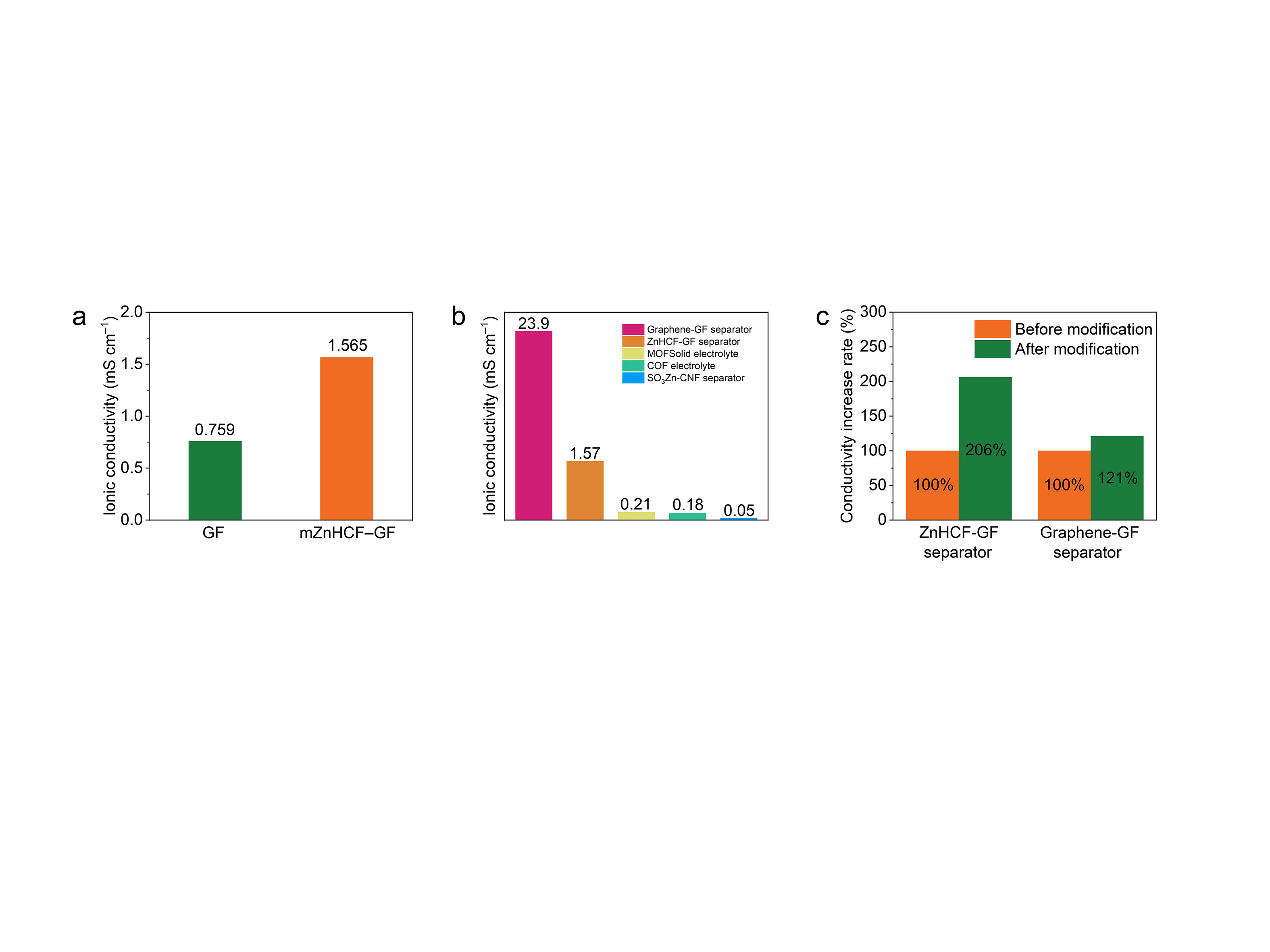
**

**Figure S15.** a) The conductivity ($\sigma$) of Ti|GF|Ti and Ti|mZnHCF–GF|Ti cell. b) Comparison of ionic conductivity of different research work.^[7-10]^

In the Ti|GF|Ti cell, the thickness of the GF separator is 0.351 mm, and the cell resistance is 26.26 Ω. As for Ti|mZnHCF–G|Ti cell, the thickness of the mZnHCF–GF separator is 0.37 mm, and the cell resistance is 13.43 Ω. The effective contact area between the electrode and the separator is 1.76 cm^−2^ for both cells with different separators. The conductivities were calculated from *Eq*. S1. It is important to note that the thickness is measured after assembling the battery at a fixed pressure and then disassembling the battery to avoid the effects of the glass fiber separator's own expansion and finger pressure.


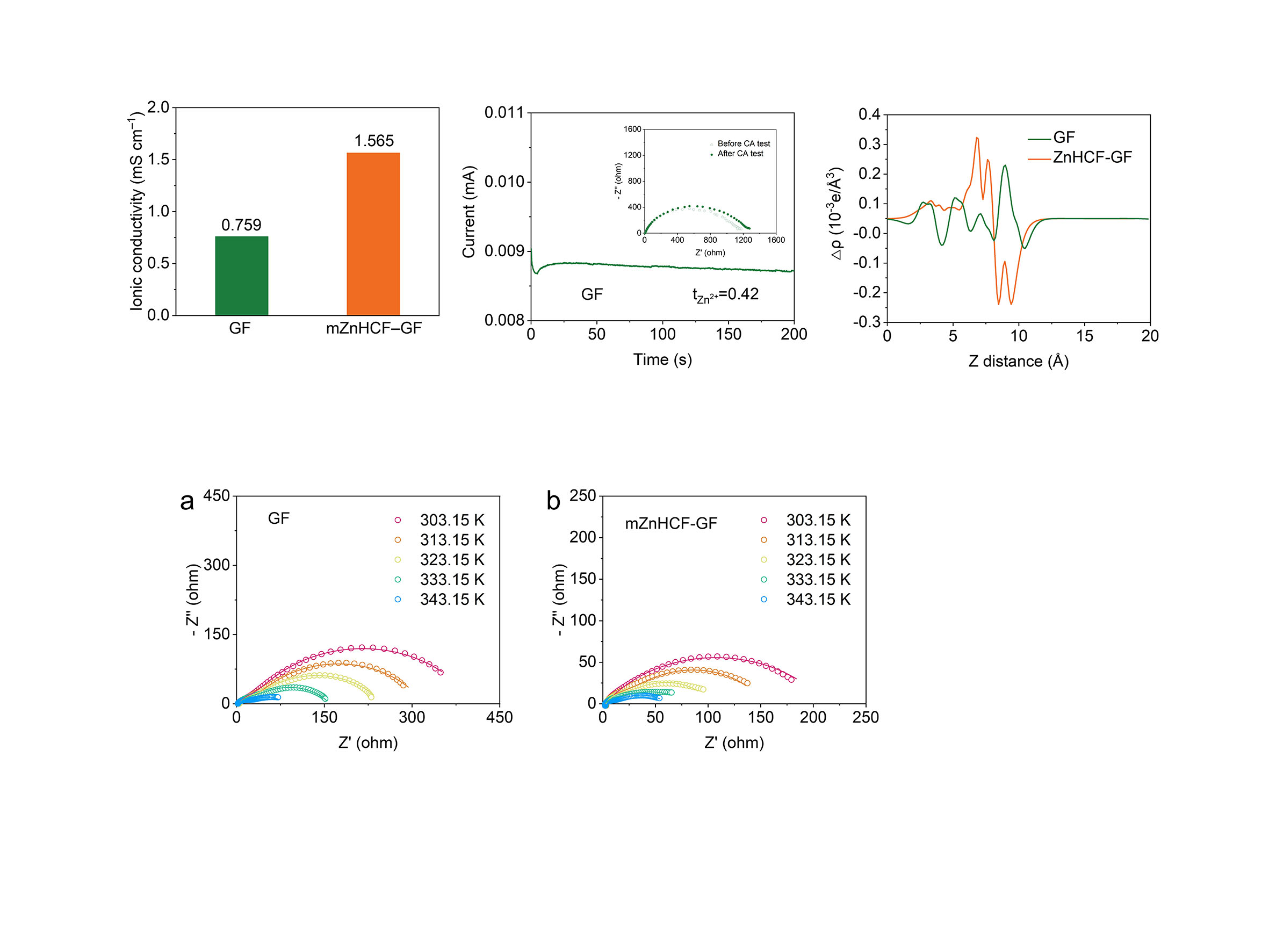


**Figure S16.** EIS spectra at different temperatures of the Zn|GF|Zn and Zn|mZnHCF–GF|Zn cells.

**
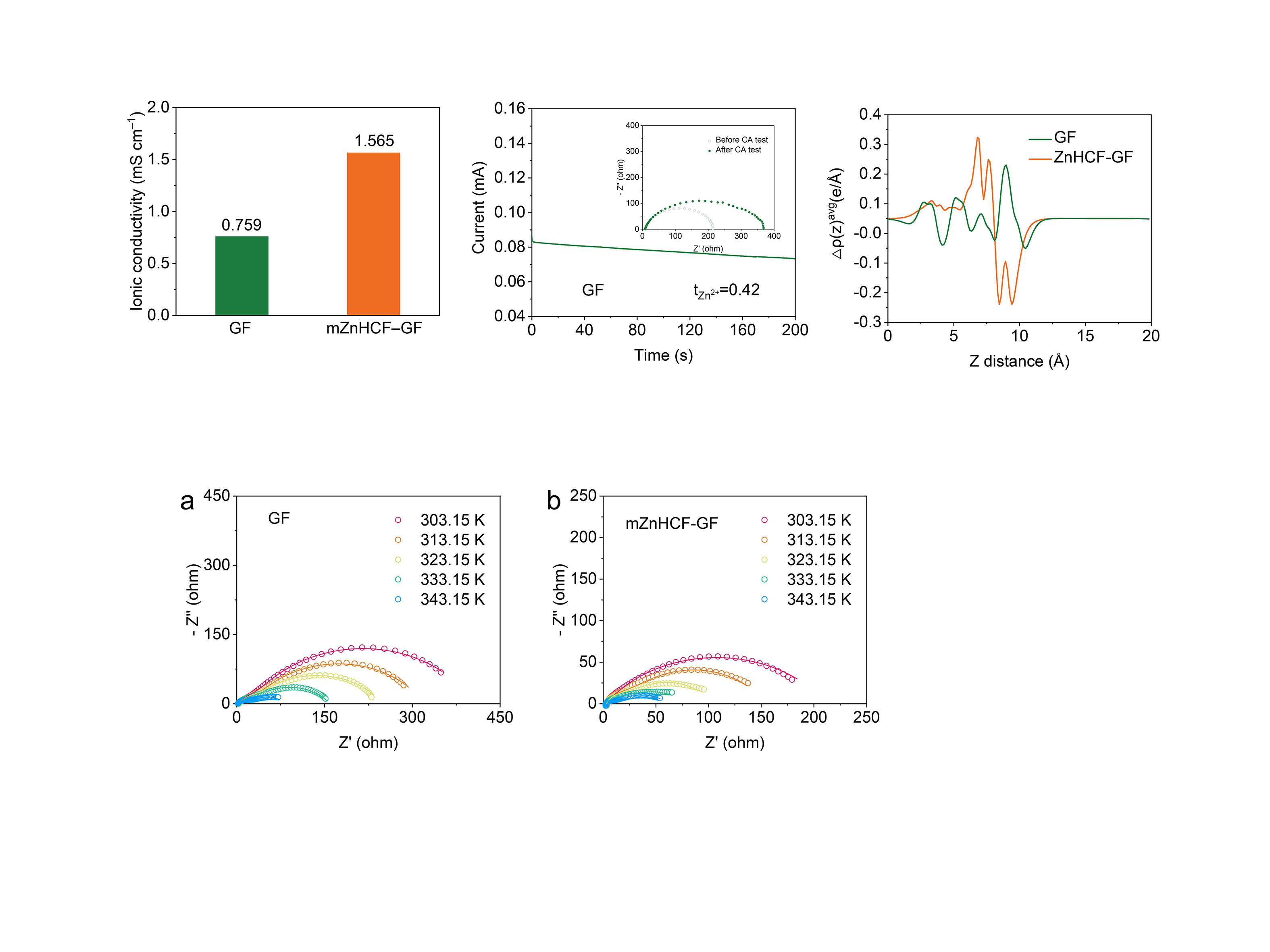
**

**Figure S17.** CA test of Zn|GF|Zn cell. Inset: Corresponding Nyquist plots before and after the CA test.


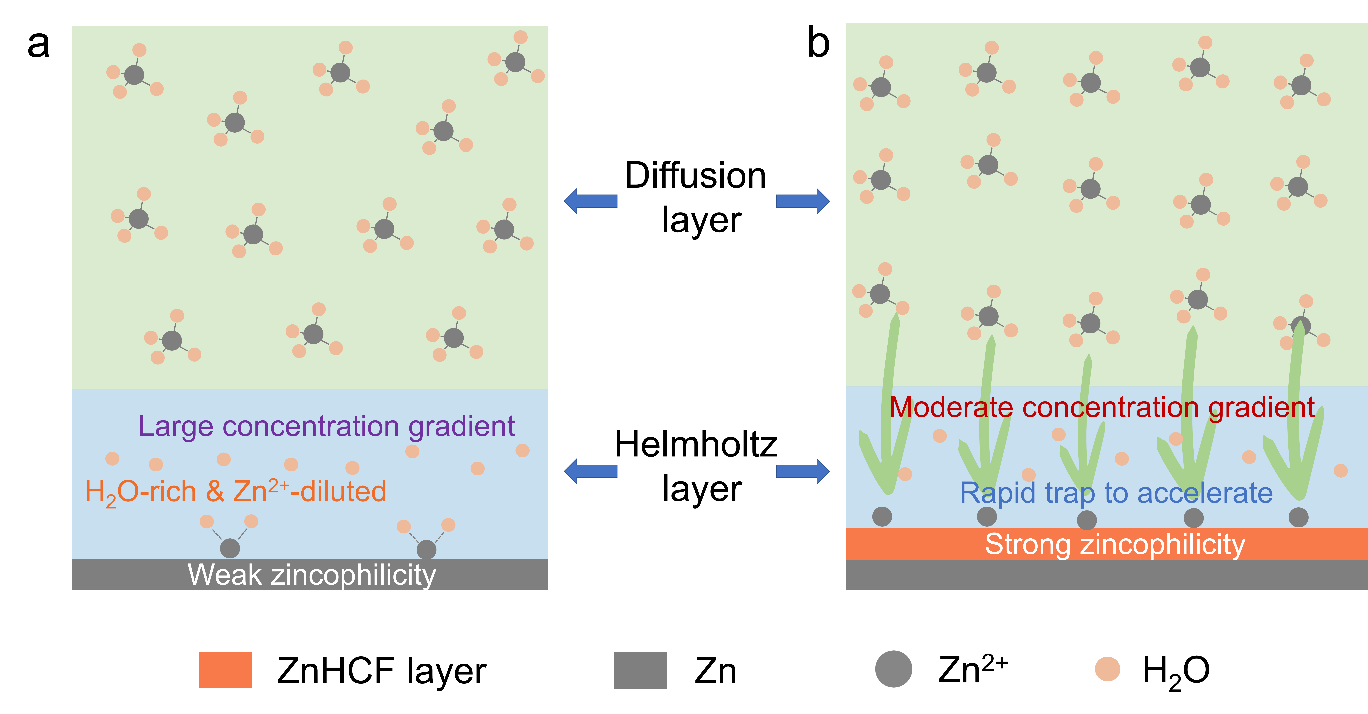


**Figure S18.** Diagram of Zn^2+^ acceleration and concentration gradient regulation mechanism.


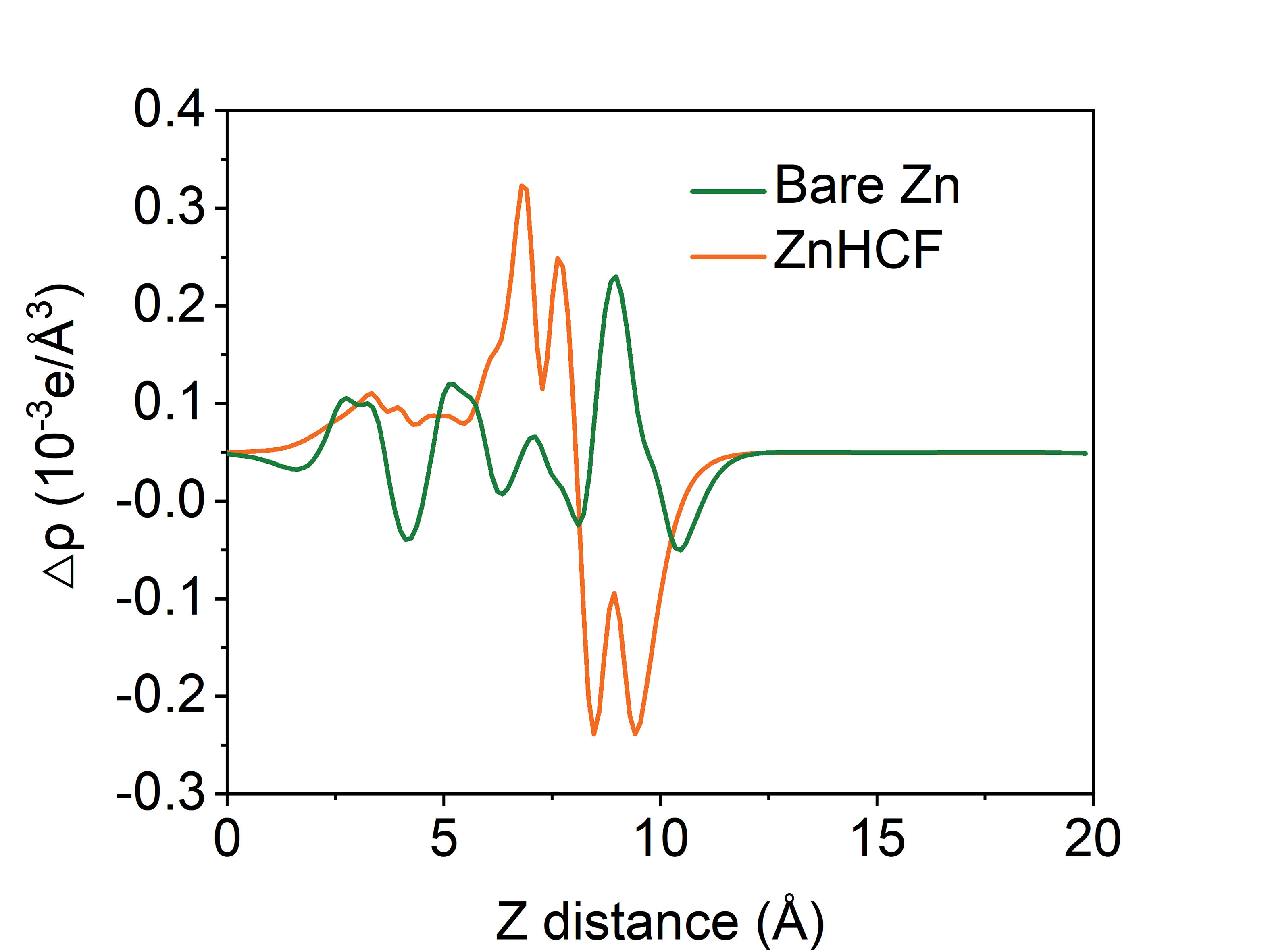


**Figure S19.** Differential charge densities calculation results of Zn atom between the Zn (002) plane and ZnHCF (116) plane. The horizontal axis represents the distance between the Zn atoms and the sites on the Zn (002) /ZnHCF (116) crystal plane along the Z-axis direction, while the vertical one corresponds to the corresponding average differential charge density, respectively.


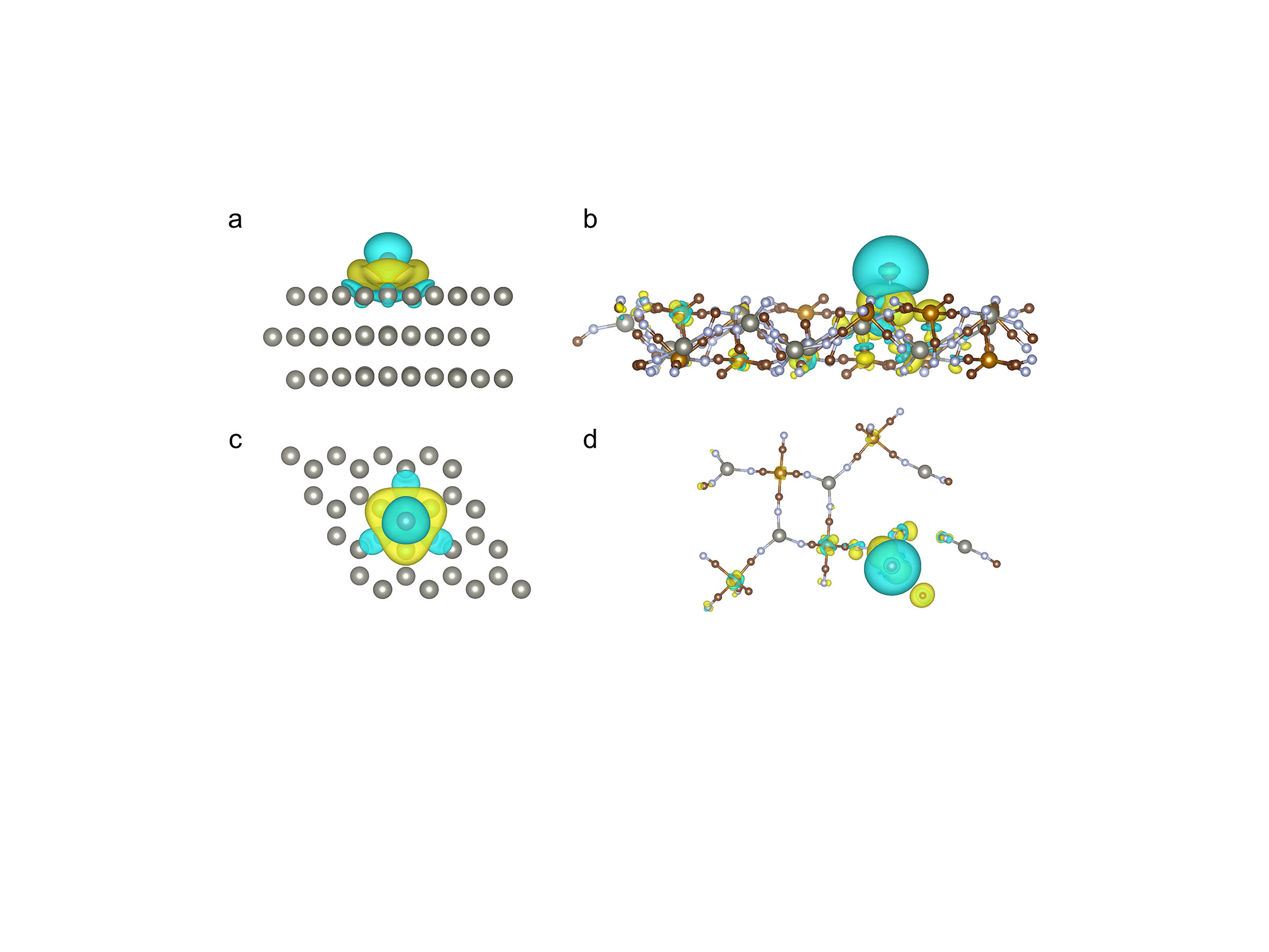


**Figure S20.** a-b) Front view and c-d) top view of differential charge density distributions of Zn atom between the Zn (002) plane and ZnHCF (116) plane.

**
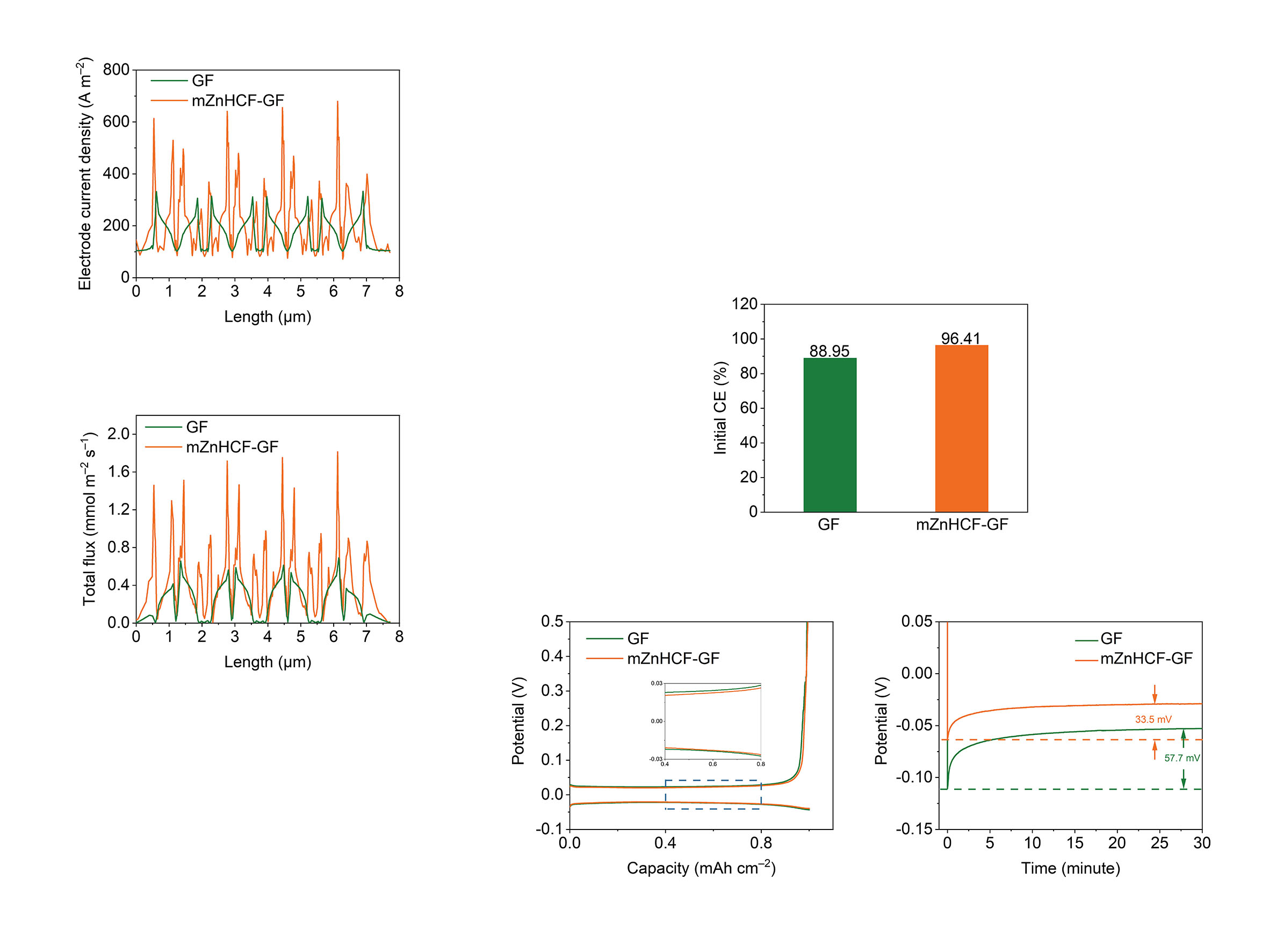
**

**Figure S21.** The electrode surface current density coupled to GF and mZnHCF–GF separator.

**
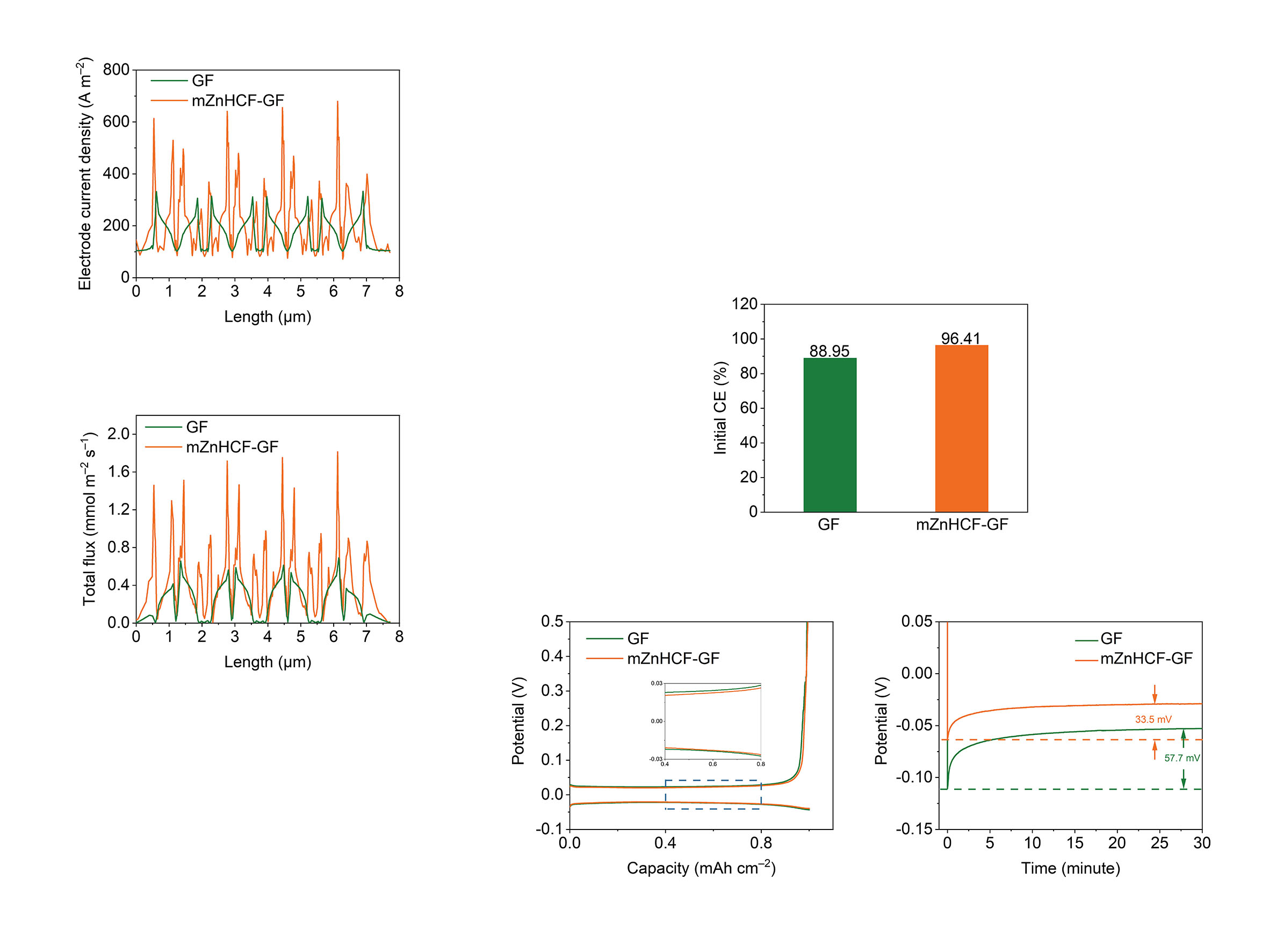
**

**Figure S22.** The total Zn^2+^ flux of electrode surface coupled to GF and mZnHCF–GF separator.

**
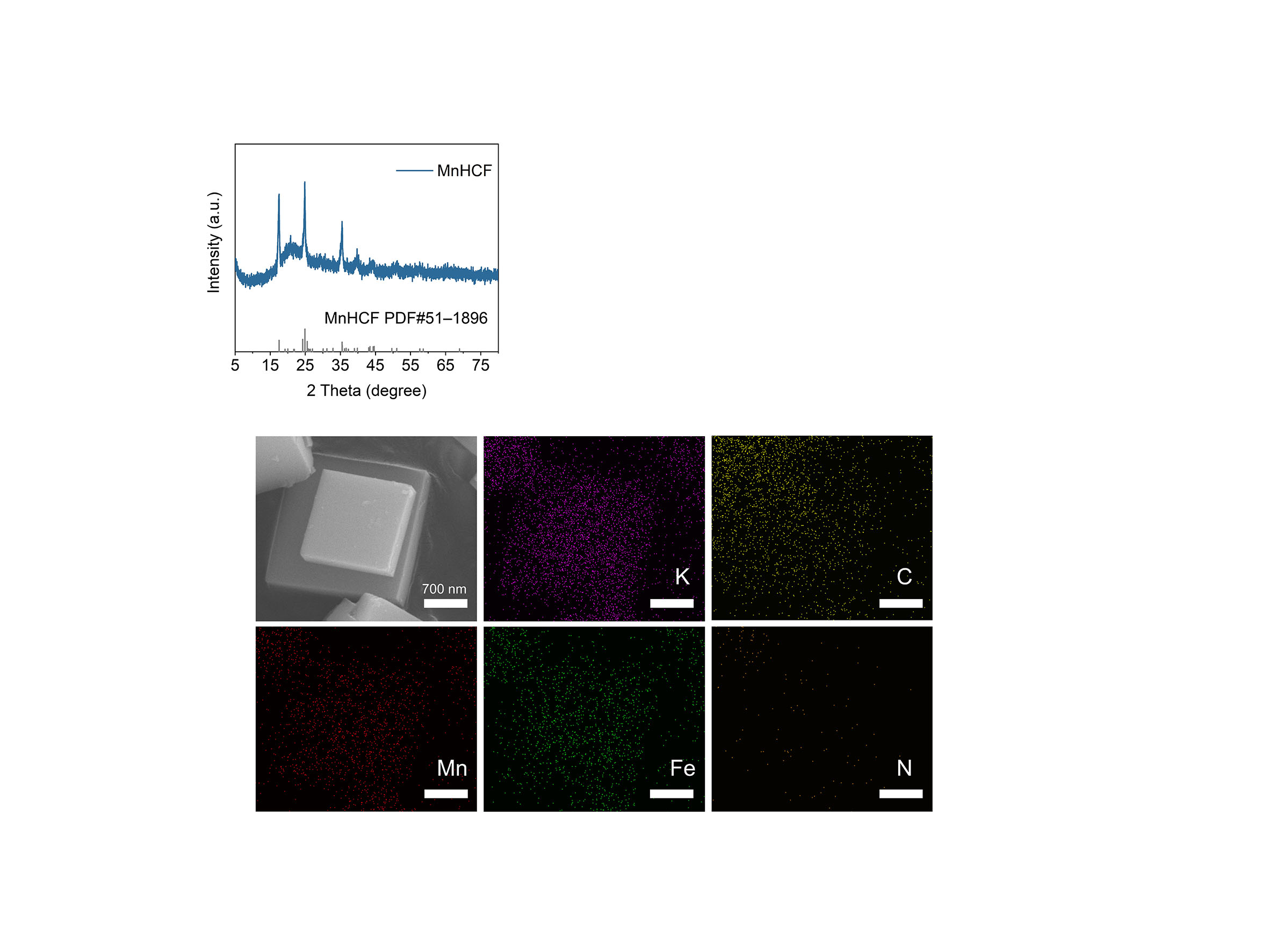
**

**Figure S23.** XRD pattern of the as-prepared MnHCF powder.

**
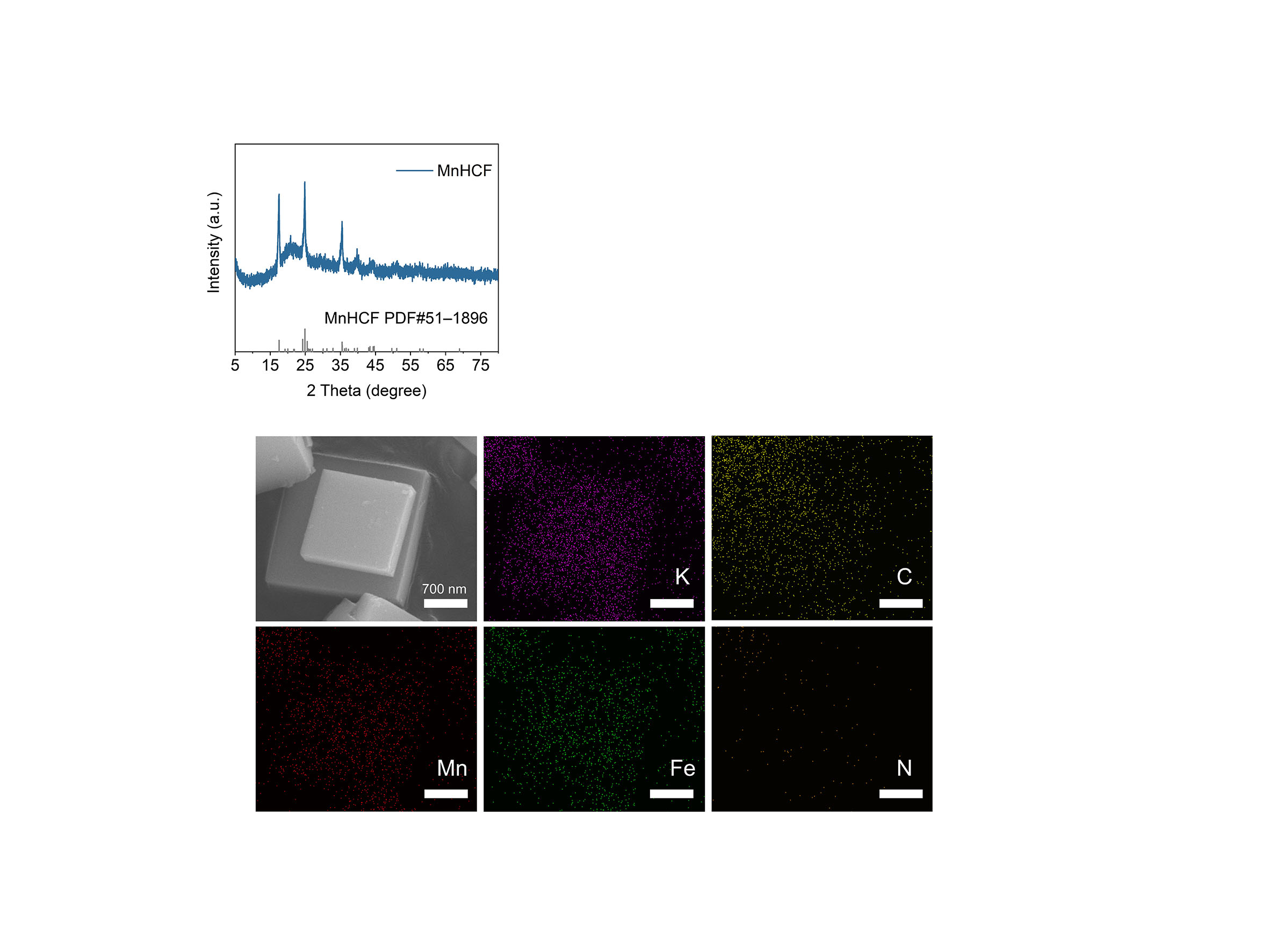
**

**Figure S24.** SEM images of the as-prepared MnHCF powder and the corresponding EDS image.

**
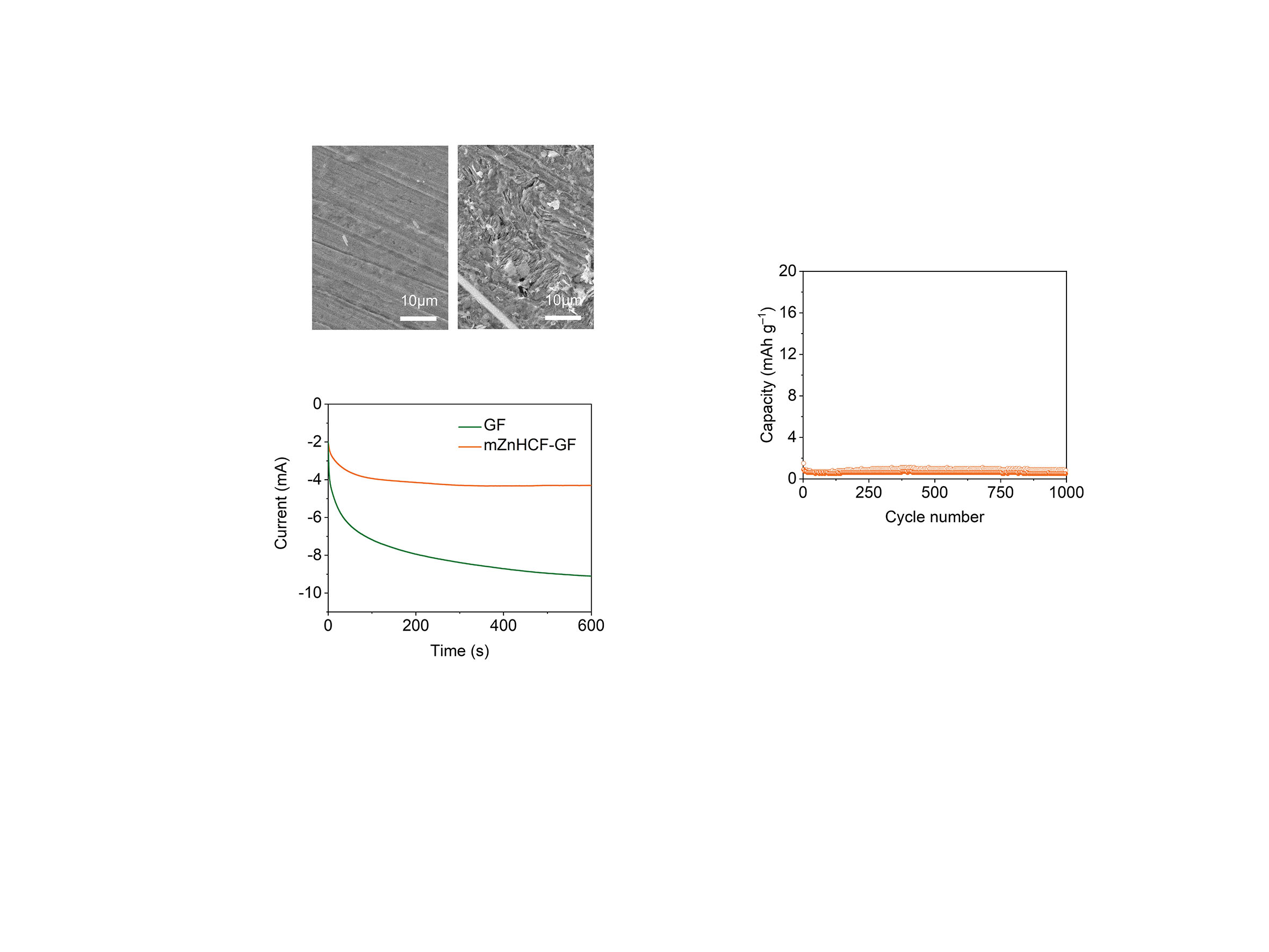
**

**Figure S25.** Cycling performances of Zn|mZnHCF–GF|Ti battery at 0.1 A g^−1^ (assume that the active material is 1mg).

**
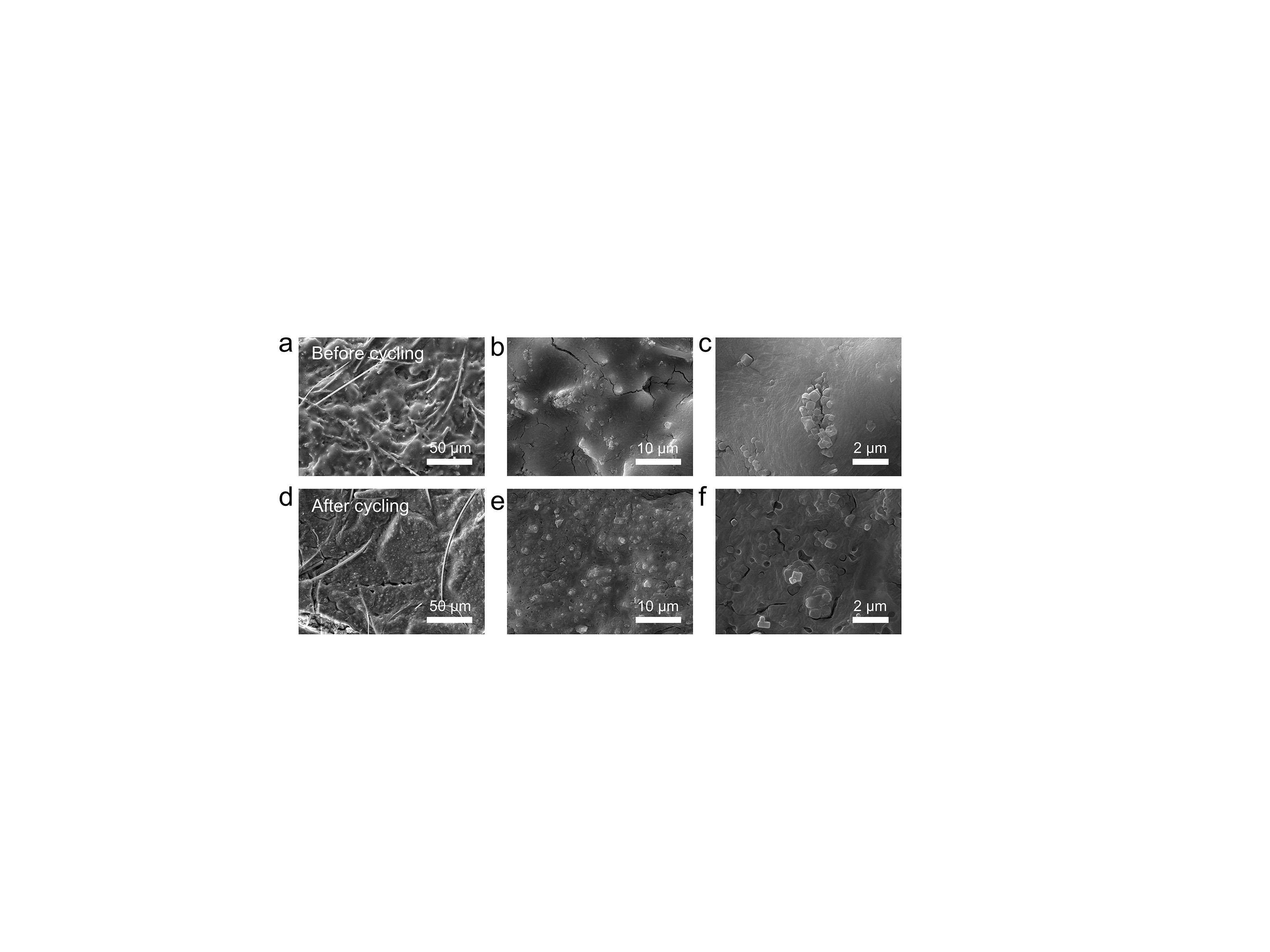
**

**Figure S26.** SEM images of the mZnHCF–GF separator a-c) before and d-f) after cycling in the ZnSO_4_ electrolyte.

**
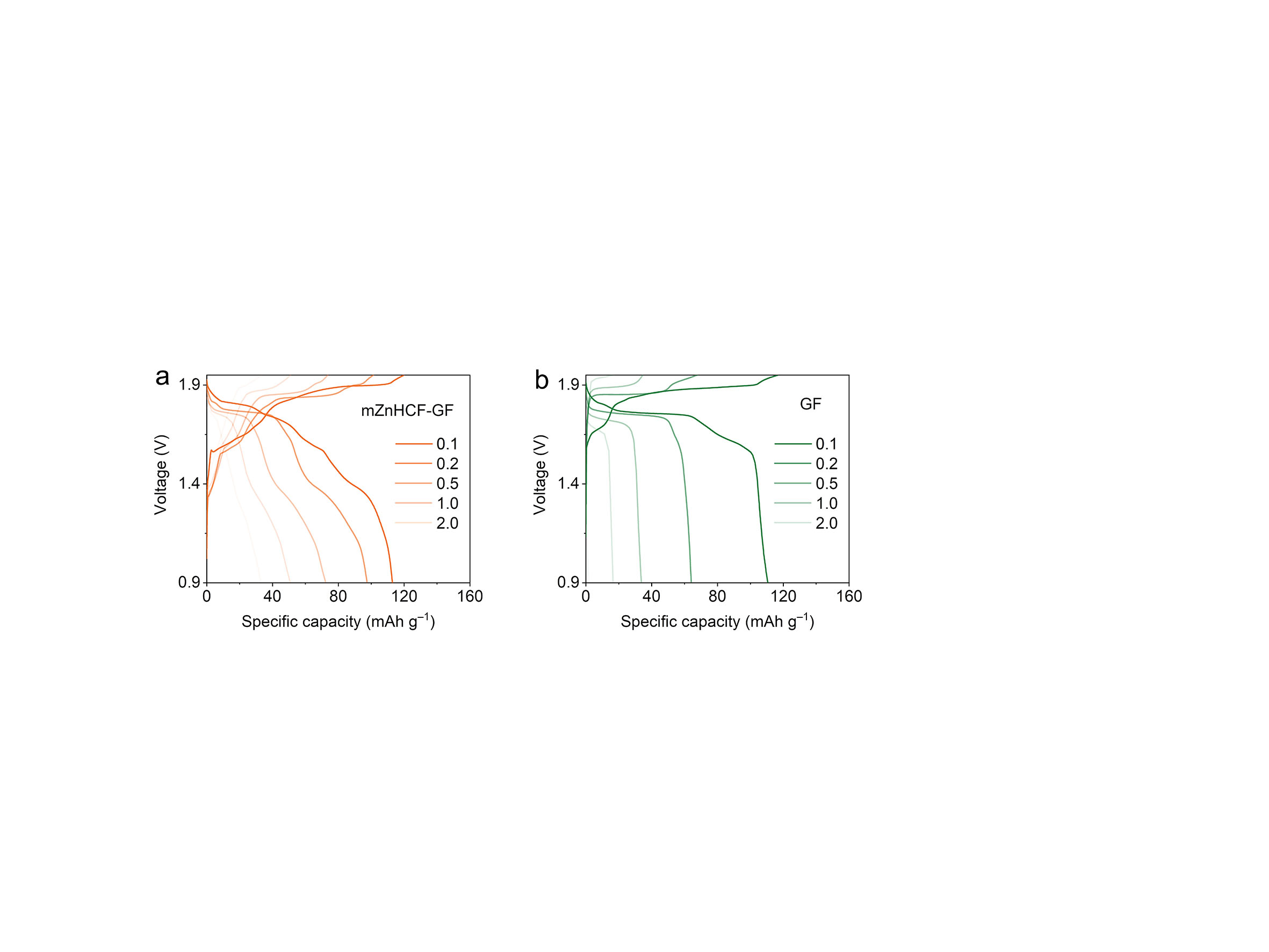
**

**Figure S27.** Charge/discharge curves of Zn||MnHCF batteries equipped with a) mZnHCF–GF and b) GF separator at various current densities.


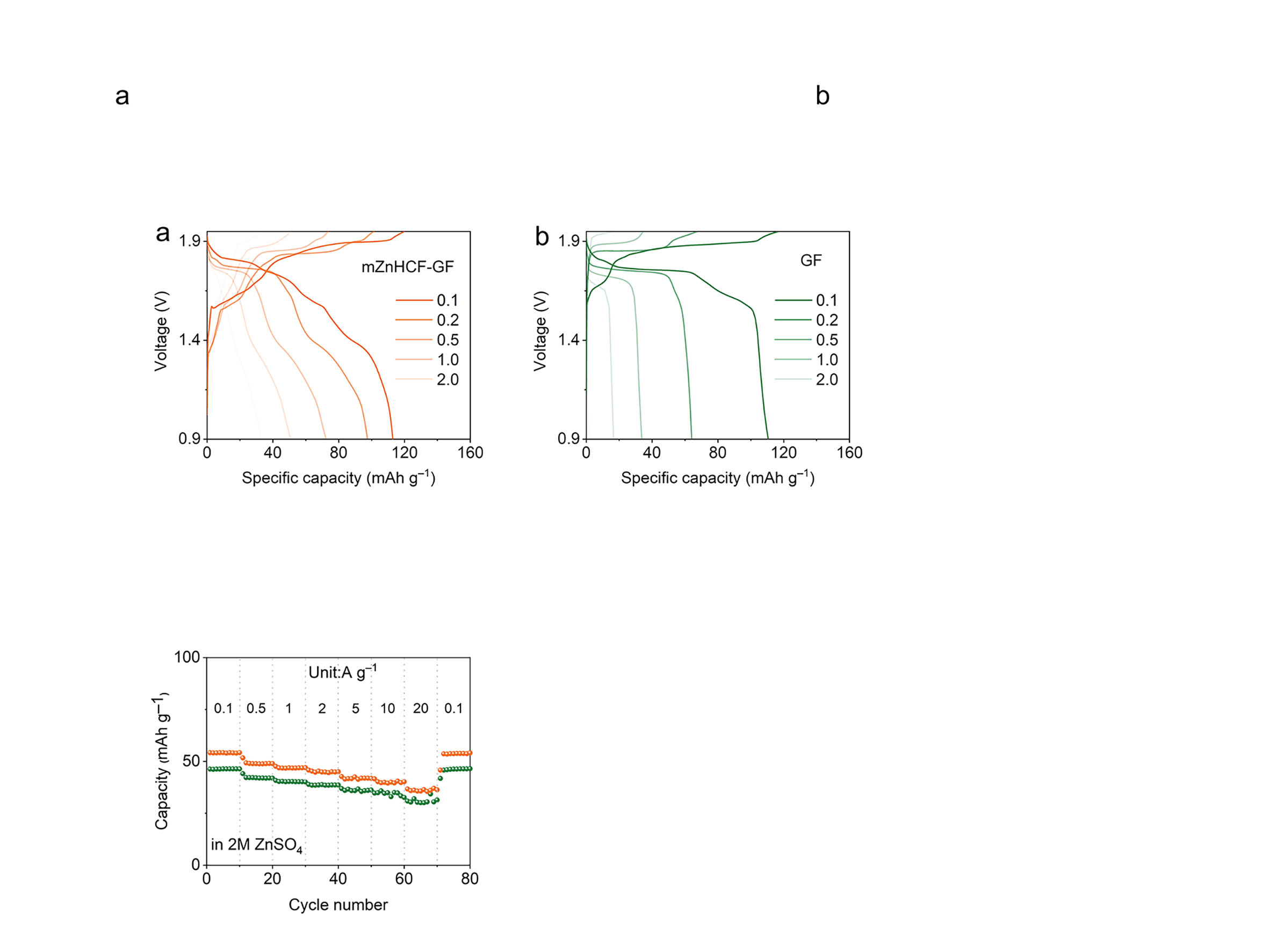


**Figure S28.** Rate performances for Zn||AC capacitors based on different separators.

Table S1. Comparison of electrochemical properties of different modified separators.

| **Separator modification strategy** | **Test conditions** | | **Lifespan** | ***Ref.*** |
| --- | --- | --- | --- | --- |
|  | Current density  (mA cm^−2^) | Capacity density  (mAh cm^−2^) |  |  |
| mZnHCF–GF separator | 2 | 1 | 2700 h | This work |
|  | 10 | 2 | 1770 h |  |
| MOF/rGO Janus separator | 2 | 1 | 500 h | [43] |
| BCM separator | 2 | 1 | 445 h | [37] |
| PVDF@GF separator | 10 | 1 | 520 h | [44] |
| Filter membrane separator | 10 | 1 | 390 h | [32] |
| UiO–66–GF–2.2 separator | 2 | 1 | 1650 h | [21] |
| CAN–2 separator | 10 | 2.5 | 1000 h | [41] |
| PVDF@PDA separator | 2 | 1 | 225 h | [31] |
| PNGF separator | 10 | 4 | 400 h | [40] |
| CF separator | 2 | 4 | 900 h | [20] |
| GF@SM separator | 2 | 2 | 500 h | [39] |
| GF/PBPT separator | 2 | 2 | 600 h | [38] |
| FCNF separator | 10 | 2 | 620 h | [17] |
| Graphene Carpets Janus separator | 10 | 1 | 600 h | [36] |
| Janus Ag Nanowires/Bacterial Cellulose separator | 2 | 1 | 2400 h | [19] |
| Zr–CNF separator | 10 | 2 | 705 h | [35] |
| DIE separator | 10 | 2.5 | 1600 h | [18] |
| MXene–GF separator | 10 | 10 | 120 h | [34] |
| CG separator | 2 | 1 | 1750 h | [33] |
| COF@GF separator | 10 | 1 | 1580 h | [42] |

Table S2. The average specific capacity of Zn||MnHCF battery at various current densities.

|  | 0.1 A g^−1^ | 0.2 A g^−1^ | 0.5 A g^−1^ | 1.0 A g^−1^ | 2.0 A g^−1^ | 0.1 A g^−1^ |
| --- | --- | --- | --- | --- | --- | --- |
| Zn\|mZnHCF–GF\|MnHCF | 112 mAh g^−1^ | 98 mAh g^−1^ | 72 mAh g^−1^ | 51 mAh g^−1^ | 32 mAh g^−1^ | 118 mAh g^−1^ |
| Zn\|GF\|MnHCF | 103 mAh g^−1^ | 63 mAh g^−1^ | 34 mAh g^−1^ | 16 mAh g^−1^ | 2 mAh g^−1^ | 88 mAh g^−1^ |

Table S3. Fitted data for EIS of Zn||MnHCF battery.

|  | R_s,_ Ω | R_CT,_ Ω |
| --- | --- | --- |
| Zn\|mZnHCF–GF\|MnHCF | 1.83 | 124 |
| Zn\|GF\|MnHCF | 4.21 | 557 |

**References**

[1] P. E. Blochl, *Phys. Rev. B* **1994**, *50*, 17953-17979.

[2] G. Kresse, J. Furthmüller, *Phys. Rev. B* **1996**, *54*, 11169.

[3] P. Hohenberg, W. Kohn, *Physical Review* **1964**, *136*, B864-B871.

[4] W. Kohn, L. J. Sham, *Physical Review* **1965**, *140*, A1133-A1138.

[5] J. P. Perdew, K. Burke, M. Ernzerhof, *Phys. Rev. Lett.* **1996**, *77*, 3865.

[6] H. J. Monkhorst, J. D. Pack, *Phys. Rev. B* **1976**, *13*, 5188-5192.

[7] X. Ge, W. Zhang, F. Song, B. Xie, J. Li, J. Wang, X. Wang, J. Zhao, G. Cui, Adv. Funct. Mater. **2022**, *32*, 2200429.

[8] X. Zhang, J. Li, K. Qi, Y. Yang, D. Liu, T. Wang, S. Liang, B. Lu, Y. Zhu, J. Zhou, *Adv. Mater.* **2022**, 2205175.

[9] S. Park, I. Kristanto, G. Y. Jung, D. B. Ahn, K. Jeong, S. K. Kwak, S.-Y. Lee, *Chemical Science* **2020**, *11*, 11692-11698.

[10] Z. Wang, J. Hu, L. Han, Z. Wang, H. Wang, Q. Zhao, J. Liu, F. Pan, *Nano Energy* **2019**, *56*, 92-99.
